# Differential Lipid Profiles of Normal Human Brain Matter and Gliomas by Positive and Negative Mode Desorption Electrospray Ionization – Mass Spectrometry Imaging

**DOI:** 10.1371/journal.pone.0163180

**Published:** 2016-09-22

**Authors:** Alan K. Jarmusch, Clint M. Alfaro, Valentina Pirro, Eyas M. Hattab, Aaron A. Cohen-Gadol, R. Graham Cooks

**Affiliations:** 1 Department of Chemistry and Center for Analytical Instrument Development, Purdue University, West Lafayette, Indiana, United States of America; 2 Department of Pathology and Laboratory Medicine, University of Louisville School of Medicine, Louisville, Kentucky, United States of America; 3 Department of Neurological Surgery, Indiana University School of Medicine, Indianapolis, Indiana, United States of America; West China Second Hospital, Sichuan University, CHINA

## Abstract

Desorption electrospray ionization—mass spectrometry (DESI-MS) imaging was used to analyze unmodified human brain tissue sections from 39 subjects sequentially in the positive and negative ionization modes. Acquisition of both MS polarities allowed more complete analysis of the human brain tumor lipidome as some phospholipids ionize preferentially in the positive and others in the negative ion mode. Normal brain parenchyma, comprised of grey matter and white matter, was differentiated from glioma using positive and negative ion mode DESI-MS lipid profiles with the aid of principal component analysis along with linear discriminant analysis. Principal component–linear discriminant analyses of the positive mode lipid profiles was able to distinguish grey matter, white matter, and glioma with an average sensitivity of 93.2% and specificity of 96.6%, while the negative mode lipid profiles had an average sensitivity of 94.1% and specificity of 97.4%. The positive and negative mode lipid profiles provided complementary information. Principal component–linear discriminant analysis of the combined positive and negative mode lipid profiles, via data fusion, resulted in approximately the same average sensitivity (94.7%) and specificity (97.6%) of the positive and negative modes when used individually. However, they complemented each other by improving the sensitivity and specificity of all classes (grey matter, white matter, and glioma) beyond 90% when used in combination. Further principal component analysis using the fused data resulted in the subgrouping of glioma into two groups associated with grey and white matter, respectively, a separation not apparent in the principal component analysis scores plots of the separate positive and negative mode data. The interrelationship of tumor cell percentage and the lipid profiles is discussed, and how such a measure could be used to measure residual tumor at surgical margins.

## Introduction

Ambient ionization mass spectrometry (MS) has the potential to improve tissue diagnosis and influence outcomes in patients undergoing surgical removal of cancer. Ambient ionization MS provides the opportunity to study biopsied tissue rapidly (seconds to minutes) and with minimal sample preparation [1]. Currently, pathologic diagnosis of gliomas, the most common malignant brain tumor, is performed upon formalin-fixed surgical biopsies via histopathology. However, this cannot be done on a timescale amenable to surgical guidance. Frozen tissue histopathology is performed as an alternative to provide the surgeon information but can take upwards of 20 minutes per biopsy. Methodologies for rapid intraoperative molecular diagnosis are sparse, with noteworthy ongoing interest in Raman spectroscopy [2] and fluorescence [3]; mass spectrometry, too, may meet this need. Several versions of ambient ionization MS have been tested for clinical applications. They include rapid evaporative MS for in vivo analysis of liver, lung, and colorectal cancers [4], desorption electrospray ionization (DESI) for detection of MRI contrast agents within tumors [5] and for analysis of frozen tissue sections, and substrate-based ambient ionization analysis of cancerous tissues; e.g., probe electrospray ionization [6] and touch spray [7,8].

DESI-MS uses a spray of charged solvent droplets to impact a surface and form a thin film in which analyte molecules dissolve. Subsequent droplet impacts release charged microdroplets from the surface and produce gas phase ions in the mass spectrometer [9,10]. DESI-MS can be performed on surfaces in an imaging mode, providing chemical information across two spatial dimensions [11]. Imaging (DESI-MSI) is typically performed in a line-by-line fashion by continuously scanning the DESI impact spot laterally across the sample in the x-dimension, and then stepping a defined distance in the y-dimension, and repeating this process. When performed in the full scan mode, every pixel in an MS image contains a mass spectrum that spans a user defined *m/z* range for a particular ionization mode (e.g., positive or negative ion mode). DESI-MSI spectra of biological tissues commonly detect metabolites and fatty acids in the lower *m/z* range (~50–300) and membrane lipids (such as phospholipids and sphingomyelins) in the higher *m/z* range (~700–1000).

Lipids serve many physiological and structural functions and are increasingly being considered as disease markers in cancer. Previous studies of prostate [7], bladder [12], kidney [13], breast [14], lymphoma [15], and gastrointestinal cancer [16], amongst others, have demonstrated that DESI-MS lipid profiles (*m/z* values and corresponding relative ion abundances) in combination with multivariate statistics, allow differentiation of cancer from normal tissue as corroborated by traditional histopathologic diagnosis. Further, these lipid profiles are not prone to degradation in the native atmosphere over the timescale of analysis, and have so far proven to be sufficiently reproducible, provided the same DESI conditions and solvents are utilized [17]. Lipid profiles acquired by DESI-MS analysis of human brain tumors have been studied previously; in particular those acquired in the negative ionization mode have been exploited for differentiating normal tissue from diseased [18] as well as for exploring the chemical differences among glioma subtypes, grades, and tissues differing in tumor cell concentrations (i.e., relative percentage of tumor cells compared with normal cells) [19,20]. Differences in the positive ion mode DESI mass spectra between different glioma grades and subtypes were previously noted, but analysis of normal parenchyma and differentiation from diseased tissue using multivariate statistics was not undertaken [21].

Recent studies of oncometabolites in human brain tumors have demonstrated the usefulness of considering low molecular weight metabolites. Notable examples are 2-hydroxyglutaric acid (2-HG) [22] and N-acetyl-aspartic acid (NAA) [18]. When the negative mode lipid profiles and additional sources of chemical information were used together, via data fusion, improved differentiation of cancer from normal brain parenchyma (i.e., grey and white matter) was obtained [18].

This study is founded on the hypothesis that the positive ion lipid profile obtained with DESI-MSI will provide complementary chemical information and, in combination with the negative ion lipid profile, improve differentiation of normal brain parenchyma and glioma. This manuscript reports sequentially acquired positive and negative ion mode DESI-MSI of tissue sections from 39 human subjects. Multivariate statistical analysis performed upon regions of interest (ROI) revealed that the positive and negative ion mode data contained differentiating information. Further, the information in the positive ion mode was nearly equivalent to the negative ion mode in regards to sensitivity and specificity. This work serves to establish the diagnostic potential of DESI-MS information in both the positive and negative ion modes, either or both of which can be transferred to intrasurgical measurements.

## Material and Methods

Banked frozen tissue specimens from 39 human subjects, obtained from the Biorepository of Methodist Research Institute (Indianapolis, IN, USA), were studied in accordance with Purdue IRB protocol (#1410015344). Specimens were cryosectioned (15 μm thickness) and thaw mounted onto glass microscope slides. All tissue sections were stored at –80°C prior to analysis. DESI-MSI was performed using a linear ion trap mass spectrometer, LTQ (Thermo Fisher Scientific, San Jose, CA USA). Dimethylformamide-acetonitrile (1:1 *v/v*) was used for DESI-MSI to preserve tissue morphology for subsequent pathology [23]. MS images at a lateral spatial resolution of 250 μm were collected in a series of rows by coordinating linear motion of the moving stage with MS duty cycle. Each tissue section was imaged twice, initially in the positive ion mode and subsequently in the negative ion mode. The moving stage was reset to the origin position between images. Additional parameters of DESI-MSI, MS/MS, and high resolution MS (HRMS) can be found in the S1 File. After DESI-MSI, the tissue sections were stained (hematoxylin and eosin, H&E) and blindly evaluated by an expert pathologist. ROI were selected in MATLAB (MathWorks, Natick, MA USA) based on histopathologic review of the tissue after MS analysis; each ROI selection was the average spectrum of 1 mm (4 pixels) by 1 mm (4 pixels), which permitted accurate correlation of the spatial and chemical information. The set of tissue sections used provided 585 ROI; 32 selections were removed as they contained significant amounts of ions related to Optimal Cutting Temperature Polymer in the spectra (Figure A in S1 File). The remaining selections were examined by principal component analysis from *m/z* 700–1000; the exclusion of lower *m/z* values improved separation as the lower mass-to-charge signals (e.g., fatty acids) were more variable in intensity. Multivariate statistics was performed using MATLAB routines and is detailed in the S1 File. Tentative attributions of *m/z* values to specific lipid structures were made based on HRMS and MS/MS product ion scans. The statistical results are independent of lipid identification: only the full scan mass-to-charge value and corresponding MS abundance are considered.

## Results and Discussion

### Sequential Positive and Negative Ionization Mode DESI-MS Imaging

Lipid metabolism has been shown to be significantly different in cancerous and non-cancerous cells and tissues due to the roles of lipids in cell growth, membrane fluidity, cell adhesion, and energy production [24]. The analysis of biological specimens in both MS polarities provides greater coverage of the lipidome; i.e., the totality of lipids in a cell, tissue, or an organism. The lipids detected in this study are primarily membrane phospholipids including phosphatidylcholine (PC), phosphatidylethanolamine (PE), sphingomyelin (SM), ceramide (Cer), phosphatidylserine (PS), phosphatidylinositol (PI), and sulfatide (ST). Some classes of membrane lipids preferentially ionize in the positive ion mode (e.g., PC and SM) and others in the negative ion mode (e.g., phosphatidic acids, PI, and PS) depending largely on the functionality of the polar headgroup [25]. We hypothesize that it is advantageous to maximize the acquired lipid information by acquiring data in both polarities to characterize different disease states (e.g., cancer) in order to increase diagnostic accuracy.

Janfelt et al. recently demonstrated acquisition of positive and negative DESI-MS data from the same specimen by alternating MS polarity every other line in the imaging experiment [26]. Their solvent system (methanol-water 19:1) destroys tissue samples during analysis, precluding subsequent analysis or staining of the same tissue specimen. We modified their approach by using morphology preserving solvents (e.g., DMF-ACN [1:1]) which allowed the same tissue section to be imaged multiple times using DESI-MS and subsequently stained for histopathology. Further, the same tissue section was analyzed twice in succession, once in the positive and then in the negative ionization mode, with mass spectra from *m/z* 200–1000 being obtained from each MS image pixel. The acquisition of data in subsequent images, as opposed to line-by-line polarity switching [26], reduces problems that might arise from incomplete charge equilibration and thus spray stability is improved.

**Fig 1:**
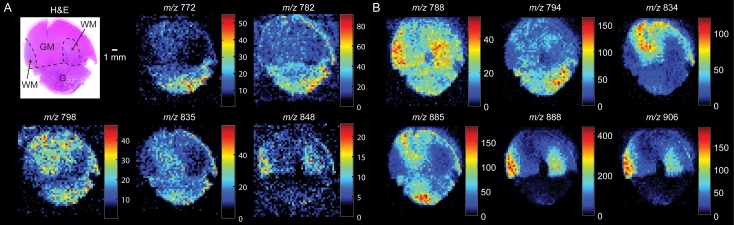
(A) Representative positive ionization mode ion images of specimen 51 and (B) representative negative mode ion images. The false-color ion images are scaled to the greatest intensity of the ion plotted in each image, the scale bar indicates the absolute MS abundance (counts) detected. Scanned H&E-stained tissue sections with regions of grey matter (GM), white matter (WM), and glioma (G) approximated based on histopathology.

Representative DESI-MS ion images for an illustrative specimen, specimen 51, are reported in Fig 1. Specimen 51 is comprised of a glioma region near the bottom of the tissue section with adjacent grey and white matter regions, determined by histopathology, and marked approximately in Fig 1A. Particular ions were relatively more abundant in different histological regions; for example *m/z* 798 ([PC 34:1 + K]^+^), 848 ([GalCer d32:2 + K]^+^), and 772 ([PE P-38:5 + Na]^+^) in the positive ion mode (Fig 1A) appear to be relatively more abundant in grey matter, white matter, and glioma, respectively. The selected ion images are a small sampling of the many ions that appear to be differentially abundant in the different histologic regions. Negative ions characteristic of grey matter (*m/z* 834, [PS 40:6 –H]^-^), white matter (*m/z* 888, [(3’-sulfo)GalCer 24:1 –H]^-^), and glioma (*m/z* 794, [PC 34:1 + Cl]^-^) were also observed at different abundances (Fig 1B). The distributions of *m/z* 834 and *m/z* 888 in grey and white matter, respectively, agree with prior DESI-MSI studies of human [18] and other mammalian brains [27], as well as other ambient ionization methods such as scanning probe electrospray ionization [28]. The combination of the positive and negative mode ion images provided visually distinct regions which matched well with histopathologic evaluation.

### Positive ionization mode DESI-MS

**Fig 2:**
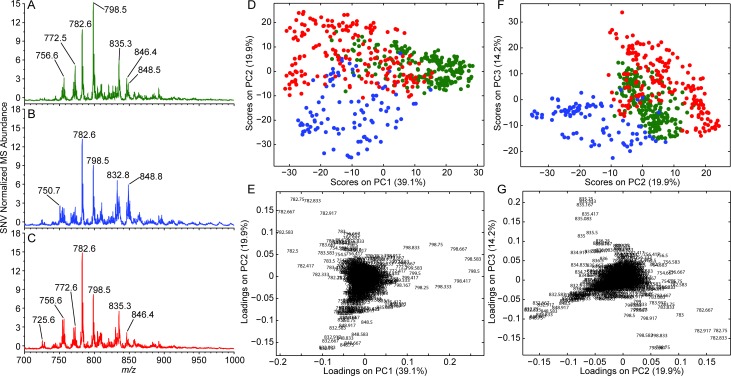
Average positive-mode DESI spectra (*m/z* 700–1000) for (A) grey matter, n = 223; (B) white matter, n = 98; and (C) gliomas, n = 185. (D and F) PCA score plots: grey matter (green), white matter (blue), and glioma (red). (E and G) PCA loading plots with ion *m/z* annotated.

The average positive mode lipid profiles for grey matter, white matter, and glioma are visually different (Fig 2A–2C, respectively). Grey matter is associated with a greater abundance of *m/z* 772 ([PC 32:0 + K]^+^) and 798 ([PC 34:1 + K]^+^). White matter appears to have greater abundances of *m/z* 750 ([GalCer d36:1 + K]^+^ and [PE P-36:2 + Na]^+^), 832 ([GalCer d32:2 + Na]^+^), and 848 ([GalCer d32:2 + K]^+^). Interestingly, gliomas appear to have greater abundances of *m/z* 754 ([PC 32:1 + Na]^+^), 756 ([PC 32:0 + Na]^+^), and 782 ([PC 34:1 + Na]^+^). Tentative identifications based on HRMS and MS/MS experiments are shown in Table A in S1 File and Figure B—Figure H in S1 File. The data show that in some cases multiple lipid species are present in a single nominal mass peak (e.g., *m/z* 750, [PE P-36:2 + Na]^+^), while other peaks are principally due to a single lipid species (e.g., *m/z* 754, [PC 32:1 + Na]^+^). The measured profiles were consistent within classes given potential biological and analytical variation. The differences between classes were typically greater than the standard deviation of the average spectrum for ions important in distinguishing the class; e.g., *m/z* 848 (Figure I in S1 File). The observed increase in total PC abundance is consistent with magic angle spinning nuclear magnetic analysis of glioma tissue [29]. Other work has suggested a role of PCs in cell proliferation rate which is frequently increased in cancer [30]. A greater abundance of SMs and ceramides in white matter versus grey matter has also been reported [31]. Plasmalogens (e.g., 750) have been reported to compose approximately 50% and 85% of the PE fraction in grey and white matter, respectively [32,33]. In addition, multiple metal adducts of the same lipid (e.g., *m/z* 782 and 798) were detected (Figure J in S1 File) and are likely due to differences in the concentration of the adducting species (e.g., Na^+^ and K^+^) and that of the analyte (e.g., PC 34:1), matrix effects, and intrinsic ionization efficiency differences. Regardless, the differences in ion abundance are consistent between classes and appear to differentiate normal parenchyma from glioma.

The positive ion mode data were analyzed by principal component analysis (PCA), an unsupervised multivariate statistical method, resulting in separation of grey matter (green points), white matter (blue points), and glioma (red points) (Fig 2). Separation of the groups in the PCA score plot of principal component 1 (PC1) vs PC2 (Fig 2D) was less clear than separation in the PCA score plot of PC2 vs PC3 (Fig 2F). The ions contributing to the observed separation in the PCA score plot are displayed in the corresponding PCA loading plots (Fig 2E and 2G). The ions displayed in the PCA loading plots recapitulate differences in the average mass spectra for grey matter, white matter, and gliomas (Fig 2A–2C, respectively). Linear discriminant analysis (LDA) was performed after compression of the variables by PCA to estimate the predictive ability of the positive ionization mode DESI-MSI data. PCA-LDA cross validation (six principal components, five deletion groups) resulted in an average sensitivity and specificity of all classes (grey matter, white matter, and glioma) of 93.2% and 96.6%, respectively. The sensitivity and specificity of each class is tabulated in Table B in S1 File. These values only provide an estimate of prediction performance; a study with a larger sample set is required for further validation but is beyond the scope of this paper.

### Negative ionization mode DESI-MS

**Fig 3:**
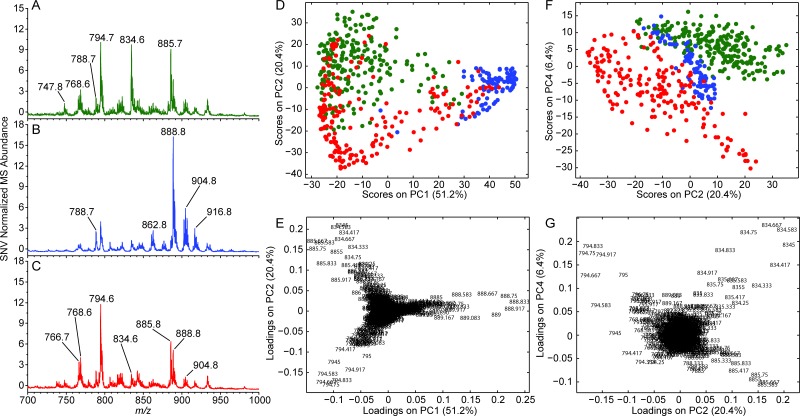
Average negative-mode DESI spectra (*m/z* 700–1000) for (A) grey matter, n = 223; (B) white matter, n = 98; and (C) gliomas, n = 185. (D and F) PCA score plots and corresponding (E and G) PCA loading plots. Grey matter, white matter, and glioma are green, blue, and red in the PCA score plot, respectively. The *m/z* of each ion is annotated in the PCA loading plot.

The presence of *m/z* 834 ([PS 40:6 –H]^–^) and 888 ([(3′-sulfo)GalCer 24:1 –H]^–^) are indicative of grey and white matter, respectively (Fig 3A and 3B). Additional predominant ions include *m/z* 788 ([PS 36:1 –H]^–^), 794 ([PC 34:1 + Cl]^–^), 885 ([PI 38:4 –H]^–^), and 906 ([(3′-sulfo)GalCer 24:0(2OH)–H]^–^). Gliomas appear to have suppressed levels of *m/z* 834 ([PS 40:6 –H]^-^) and 888 ([(3’-sulfo)GalCer 24:1 –H]^-^) relative to the grey and white matter average spectra, and therefore appear to have a visually distinct lipid profile (Fig 3C). Tentative identifications are based on previously published results [18,19]. PCA was performed on the negative ionization mode DESI-MSI data, and the resulting score and loading plots are displayed in Fig 3. The separation of grey matter (green points), white matter (blue points), and glioma (red points) as well as their lipid profiles are consistent with previous results [18] which utilized the same tissue specimens. The similarity of the results is encouraging because analyses were performed one year apart. The continuum of points observed between the main grey and white matter groupings in the PCA score plot of Fig 3D, reflect mixtures of grey and white matter that are present in normal neuroanatomy [34]. Similarly, the points which fall in between the normal parenchyma and glioma groupings represent glioma infiltration into adjacent matter which is well known [35,36]. Points (ROIs) falling in between the main groups had convoluted lipid profiles and support the presence of mixed compositions of grey, white matter, and glioma. For example, Figure K in S1 File shows the mass spectrum for a mixture of grey and white matter. In spite of the continuum between grey matter, white matter, and glioma, good separation is observed in additional PCA score plots; e.g., PC2 vs PC3 (Fig 3F). The PCA loading plots (Fig 3E and 3G) display the importance of specific ions in the separations seen in the PCA score plots.

Negative ionization mode PCA-LDA cross validation (six PCs, five deletion groups) resulted in an average sensitivity of 94.1% and specificity of 97.4%. The sensitivity and specificity of individual classes can be found in Table C in S1 File. The average values are consistent with unreported PCA-LDA results from our prior study which lie within ~5% [18]. Overall, the average positive and negative ionization mode sensitivity and specificity values are similar and appear to be equivalently useful in separating grey matter, white matter, and gliomas. The notable differences when comparing the classes individually are as follows: the sensitivity of gliomas in the positive mode was higher than the negative mode (93.0% versus 88.1%), respectively, while the sensitivity of white matter was lower (89.8%, positive mode, versus 95.9%, negative mode). The specificity of grey matter was greater in the negative mode than the positive mode (98.8% versus 92.2%); the specificity of white matter was higher in the positive mode, 99.7%, than in the negative mode, 93.9%. Comparison of the positive and negative mode cross validation results show that each mode provides complementary diagnostic information, while the average sensitivity and specificity of all classes are similar.

### Positive and Negative Mode Data Fusion

**Fig 4:**
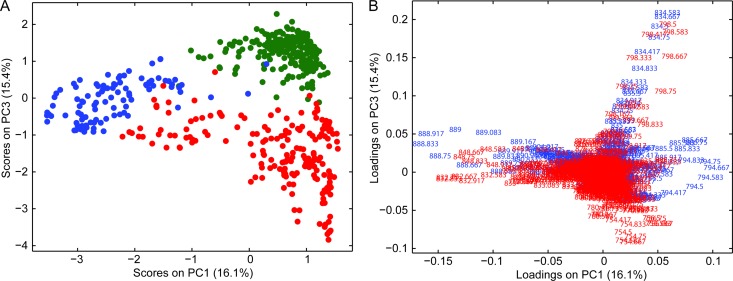
(A) Midlevel data fusion PCA score plot (*m/z* 700–1000) and (B) loading plots. Score plot symbols: Grey matter (green), n = 223; white matter (blue), n = 98; and glioma (red), n = 185. Loading plot values: negative mode (blue) and positive mode (red).

Midlevel data fusion was applied considering the first six principal components. PCA of the fused data resulted in clearer separation of grey matter, white matter, and glioma, as shown in Fig 4A. The improved separation reduced overlap between the groupings (e.g., grey matter and glioma). Separation was also noted in additional PCA score plots (Figure L in S1 File). Interestingly, the glioma class was split into two subgroups, one closely associated with the white matter group and one more closely associated with the grey matter group (approximately delineated left-to-right at a PC1 score of 0 in Fig 4A). The glioma subgroups were not apparent in the positive or negative mode lipid profiles individually or in our prior study in which data for the lipids and metabolites in the negative ion mode were fused [18]. The ROIs of each glioma subgroup were averaged and are displayed in Figure M in S1 File. The negative mode lipid profile of the grey matter–associated glioma subgroup was similar to previously reported glioma profiles [18,20] and contained neither *m/z* 834 nor 888 at any appreciable abundance. The negative mode lipid profile of the white matter–associated glioma subgroup was similar to the negative mode white matter lipid profile. The positive mode lipid profiles for the two subgroups were visually different from those of normal grey and white matter. The PCA loading plot (Fig 4B) offers a basic understanding of the correlation between positive and negative ions; vectors of similar direction indicate a positive correlation; e.g., *m/z* 798 (positive mode) and 834 (negative mode) are important in separating grey matter. Similarly, *m/z* 832 (positive mode) and 888 (negative mode) are important in separating white matter. Additional detail as to the loading value of individual ions are shown in Figure N in S1 File. The loading plot does not reveal deeper relationships (e.g., biological reason) for the correlation between ions, only that they are correlated in separating the groups. A PCA score plot using the first three PCs (S1 Video) illustrates the separation of the classes in 3D space.

PCA-LDA was performed on the fused data (six principal components, five deletions groups) and resulted in the following cross validation results (Table 1). The average sensitivity and specificity of all classes was 94.7% and 97.6%, respectively. These values are not substantially different than the positive or negative mode separately. However, the sensitivity and specificity of individual classes was improved no value falling below 90%. Fusion of the data in some cases did slightly decrease the sensitivity or specificity of individual classes compared to the positive or negative mode results; e.g., specificity of white matter: fused (95.1%), positive mode (99.7%), and negative mode (93.9%). The PCA-LDA results support that information in the positive and negative lipid profiles are complementary and aid in more confidently distinguishing grey matter, white matter, and glioma.

**Table 1:** Data fusion PCA-LDA confusion matrix with calculated sensitivity and specificity for grey matter, white matter, and glioma. The average sensitivity, 94.7%, and specificity, 97.6%, is the mean of the values for the individual classes.

		Histopathology		
		Grey matter	White matter	Glioma
DESI	Grey matter	216	2	1
	White matter	5	94	15
	Glioma	2	2	169
	Sensitivity (%)	96.9	95.9	91.4
	Specificity (%)	98.9	95.1	98.7

### DESI-MS Detection of Tumor Cell Percentage

Tumor cell percentage (TCP), previously dubbed tumor cell concentration, is a means by which to measure glioma infiltration. Its interrelationship with the lipid profiles is poorly understood except that it is partially responsible for the spread of points in the PCA score plots between groupings. The TCP, estimated via histopathology, was plotted (Figure O in S1 File); the PCA score plot indicated that the majority of the grey matter associated glioma subgroup was of medium to high TCP (regions defined as 33%< x <67% and >67%, respectively) whereas the white matter associated glioma subgroup was comprised of ROIs with low (<33%) to medium TCP. The grey matter associated glioma subgroup was better separated than the white matter associated glioma subgroup from their respective normal groupings, potentially the effect of a relatively higher TCP. Further, tumor grade and TCP are not completely independent as gliomas of higher grade (WHO III and IV) tend to have greater TCP; differences between tumor grades, plotted in Figure O in S1 File, cannot be commented upon in this study due to the low number of low grade gliomas (WHO I and II).

The complex nature of the molecular diagnosis of gliomas is illustrated in specimen 65 which contained regions of different TCP (40–60%). Illustrative ROI mass spectra are displayed in Figure P in S1 File. The positive and negative spectra of the ROI associated with 60% TCP is less reminiscent of white matter than the spectra from the 40% TCP region. The ROIs of specimen 65 were projected onto the fusion PCA score plot (Figure Q in S1 File) using a technique similar to that described in Bagnaso et al. [37] The ROIs associated with lower TCP in specimen 65 were projected within the white matter group while the ROIs associated with 60% TCP were projected within the white matter–associated glioma subgroup. Similarly, specimen 24, a heterogeneous tissue section, was comprised of a higher TCP glioma region (~80%) and a lower density tumor (10% TCP) in mostly grey matter (one small area of white matter was also present) (Figure R in S1 File). The ROI associated with the 80% TCP region of specimen 24 lay within the grey matter associated glioma subgroup, while the ROI associated with the 10% TCP grey matter region lay within the grey matter group. Note, ROI 13 lay between the grey matter and white matter groups and corresponds to a selection near a grey and white matter boundary.

Lipid changes associated with glioma, detected in the positive and negative mode lipid profiles, appear to be too attenuated to be a useful discriminator in areas of lower tumor cell percentage. Given that the future objective of this work is to assist in surgical resection, it is necessary to measure TCP levels at the surgical resection margin which may be in the 10–40% range, although no current information exists as to the actual proportion of tumor cells at the surgical margin. Current practice determines resection completeness via contrast enhanced MRI, which is an improvement over visual inspection [38]; however, this methodology relies on uptake of radiocontrast agent, can be confounded by intracranial volume changes, and cannot be used to interrogate specific regions that the neurosurgeon finds pathologically suspicious. For glioblastoma patients, there is controversy as to the effect of completeness of tumor resection and patient survival because the infiltrative nature of glioblastoma renders total surgical resection an impossible task with current surgical techniques [39]. Even in tissues that appear histologically normal, malignant glioma cells can be isolated and cultured. Indeed, the site of tumor recurrence is often within two centimeters of the resection margin from non-enhancing areas observed in MRI [40,41]. However, studies have shown that near-total tumor resection for low grade glioma patients significantly increases patient survival and time to malignant progression [42,43]. Optimization and evaluation of the DESI-MS methodology to assist the surgeon in checking discrete areas within the operative field for residual tumor, via the determination of TCP based on DESI-MS lipid profiles, is the subject of future work.

### DESI-MS Analysis of Morphologically Effaced Tissue Sections

DESI-MSI could also be used to complement glioma diagnosis (particularly on frozen sections) as tumors commonly efface normal tissue morphology such that the background parenchyma cannot be definitively identified. For example, we found in specimen 70 that the DESI-MS data could be used to determine the background parenchyma when histopathology was inconclusive (Figure S in S1 File). Chemically, it was quite clear that the background parenchyma of specimen 70 was white matter due to the presence of *m/z* 888. Such lipid information provided by DESI-MSI could be useful in clarifying current histopathologic evaluation and potentially enhance detection of glioma infiltration in tissue sections.

## Conclusions

The serial acquisition of DESI-MS images in the positive and negative modes provided spatial and chemical information (i.e., lipid profiles) that differentiated human grey matter, white matter, and gliomas. The positive ion mode lipid profiles are reproducible and allow differentiation of grey matter, white matter, and gliomas with an average sensitivity and specificity of 93.2% and 96.6%, respectively. The inclusion of normal brain parenchyma samples and differentiation via multivariate statistics expand upon prior work which explored the differences in the positive mode lipid profile between different glioma grades and subtypes [21]. The positive ion mode lipid profiles yielded approximately the same differentiation ability as the negative ion lipid profiles. The repeatability of the acquired lipid profiles is illustrated by the similarity of the negative ion mode PCA, average mass spectra, and PCA-LDA cross validation results with that of a prior study [18]. Our hypothesis was supported as the complementary chemical information obtained from the positive and negative mode analyses slightly improved PCA separation of grey matter, white matter, and glioma when used together via data fusion. The fused data analyzed by PCA-LDA provided an average sensitivity of 94.7% and specificity of 97.6%, and the individual class values all exceeded 90%. Diagnosis of effaced glioma tissue specimens is complex, but DESI-MS lipid profiles were able to suggest, chemically, the background matter in such specimens. It is foreseeable that providing pathologists with supplemental DESI-MSI information would increase their diagnostic confidence. Regarding use in the surgical resection of gliomas, we observed the influence of glioma infiltration (assessed as TCP) on the lipid profiles and discussed the possible uses and limitations of this measurement. The limited ability of the lipid profiles to determine lower TCP emphasizes the need for further improvement to the current DESI-MS and multivariate statistical methods. One candidate solution is to combine the negative and positive ion mode lipid profiles with chemical measurements of oncometabolites, such as NAA, that distinguish normal and cancerous neural tissue with high sensitivity and specificity [44]. We envision that the intraoperative use of DESI-MS, collecting positive and negative mode lipid and metabolite data, will comprise an *ex vivo* tool to check discrete areas of tissue which are pathologically ambiguous by visual inspection, potentially augmenting current surgical practice.

## Supporting Information

S1 File **Table A.** HRMS and MS/MS data for selected ions detected in positive mode DESI-MSI of brain tissue and glioma specimens. Exact mass measurements were searched in the METLIN database (https://metlin.scripps.edu/metabo_search_alt2.php). MS/MS spectra were examined for expected losses from membrane lipid head groups such as 59 (trimethylamine) and 183 (phosphocholine) for PCs and SMs [45,46]; 162 (dehydrated C_6_ sugar), 180 (C_6_ sugar) for GalCer [47]; 43 and 141 (phosphoethanolamine) for PE [48,49]. Molecular species are consistent with previously published results in which lipid profiles from a small set of human astrocytoma specimens were obtained [21]. Plasmalogens were also detected at certain *m/z* values and supported with MS/MS experiments. **Table B.** Positive ion mode PCA-LDA confusion matrix (six principal components, five deletion groups) for grey matter, white matter, and glioma with calculated sensitivity and specificity for each class. **Table C.** Negative ion mode PCA-LDA confusion matrix (six principal components, five deletion groups) for grey matter, white matter, and glioma with calculated sensitivity and specificity for each class. **Figure A.** (A) Negative-mode DESI-MS spectra of ROI removed due to OCT signal (B) detected in the positive-mode. OCT is a polymeric material, principally polyvinyl alcohol, which is readily ionized in the positive ion mode, easily recognized by polymeric peaks separated by 22 mass-to-charge units (e.g., *m/z* 708.7, 730.7, and 752.7; *m/z* 715.1 and 737.1; *m/z* 722.7 and 744.7). **Figure B.** High resolution mass spectrum obtained from specimen CGY, composed of normal white and grey matter, in the positive ion mode A) *m/z* range 748–811. B) *m/z* range 828–852. **Figure C.** (A) MS^2^ product ion spectrum of *m/z* 750. (B) MS^3^ product ion spectrum of *m/z* 588 ([GalCer(d36:1) + Na– 162]^+^. (C) MS^3^ product ion spectrum of *m/z* 707 ([PE(P-36:2) + Na– 43]^+^. (D) MS^2^ product ion spectrum of *m/z* 772. (E) MS^3^ product ion spectrum of *m/z* 713 ([PC 32:0 + K– 59]^+^. (F) MS^3^ product ion spectrum of *m/z* 729 ([PE(P-38:5) + Na– 43]^+^. **Figure D.** (A) MS^2^ product ion spectrum of *m/z* 754. (B) MS^3^ product ion spectrum of *m/z* 695 ([PC 32:1 + Na—59]^+^). (C) MS^2^ product ion spectrum of *m/z* 756. (D) MS^3^ product ion spectrum of *m/z* 697 ([PC 32:0 + Na—59]^+^). **Figure E.** (A) MS^2^ product ion spectrum of *m/z* 782. (B) MS^3^ product ion spectrum of *m/z* 723 ([PC 34:1 + Na– 59]^+^). (C) MS^2^ product ion spectrum of *m/z* 808. (D) MS^3^ product ion spectrum of *m/z* 749 ([PC 36:2 + Na– 59]^+^). **Figure F.** (A) MS^2^ product ion spectrum of *m/z* 798. (B) MS^3^ product ion spectrum of *m/z* 739 ([PC 34:1 + K– 59]^+^). (C) MS^3^ product ion spectrum of *m/z* 755 ([PE(P-40:6) + Na—43]^+^). **Figure G.** (A) MS^2^ product ion spectrum of *m/z* 832. (B) MS^3^ product ion spectrum of *m/z* 670 ([GalCer(d32:2) + Na– 162]^+^). (C) MS^2^ product ion spectrum of *m/z* 835. (D) MS^3^ product ion spectrum of *m/z* 776. **Figure H.** (A) MS^2^ product ion spectrum of *m/z* 848. (B) MS^3^ product ion spectrum of *m/z* 686 ([GalCer(d32:2) + K—162]^+^). **Figure I.** (A–C) Selected *m/z* regions of the positive ion mode lipid profile displaying the mean (solid line) and standard deviation (dotted line and filled area) for grey matter (green), white matter (blue), and glioma (red). (D–E) Selected *m/z* regions of the negative ion mode lipid profile. The mean (solid line) and standard deviation (dotted line with filled area) are displayed for grey matter (green), white matter (blue), and glioma (red). **Figure J.** Bar graph of the SNV normalized MS abundance of (A) *m/z* 782, (B) *m/z* 798, and (C) *m/z* 794, associated with different adducts of PC 34:1 (sodium, potassium, and chloride respectively), displaying the mean and standard deviation per class: grey matter (N = 223), white matter (N = 98), and glioma (N = 200). Statistical significance indicated (Kruskal-Wallis, 95% CI) by a single asterisk (*). Overall, the differences in abundance were not predictive by themselves due to high variances, while differences in some mean values were statistically significant at 95% CI (e.g., *m/z* 782, grey matter and glioma) by Kruskal-Wallis. **Figure K.** Illustrative (A) negative and (B) positive ion mode DESI-MS spectra from a ROI of mixed grey and white matter composition. Both *m/z* 834 and 888 are abundant in the negative ion mode. Similarly, the positive mode lipid profile appears to be a combination of grey and white matter. **Figure L.** (A) Mid-level data fusion PCA score plot, PC2 vs PC3, and (B) loading plots. Score plot symbols: Grey matter (green), white matter (blue), and glioma (red). Loading plot values: negative mode (blue) and positive mode (red). **Figure M.** (A-B) Negative and positive mode average of the white matter associated glioma subgroup, respectively. (C) Negative mode average of the grey matter associated glioma subgroup. (D) Positive mode average of the grey matter associated glioma subgroup. Major ions are annotated. **Figure N.** Mid-level fusion PCA loading values of specific ions, negative mode (blue) and positive mode (red), on principal components 1–3 with *m/z* annotated. **Figure O.** (A) PCA score plot, PC1 vs PC3, for tumor cell percentage (TCP): n/a (yellow), normal grey or white matter; low (orange), <33%; medium (red), 33%< x <67%; high (dark red), >67%. (B) PCA score plot for glioma grade: n/a (blue heather), normal grey or white matter; low grade (light blue), glioma WHO grade I or II; high grade (dark blue), glioma WHO grade III or IV. **Figure P.** (A) Negative and (B) positive ion mode lipid profiles of ROI #1 with 40% TCP. (C) Negative and (D) positive ion mode lipid profiles of ROI #5 with 60% TCP. Note, that the 40% TCP spectra look more reminiscent of normal white matter while the 60% TCP spectra appear more like the white matter associate glioma subgroup (e.g., altered ratio between *m/z* 794 and *m/z* 888 in the negative ion mode and altered ratio of *m/z* 798 and *m/z* 848 in the positive mode). **Figure Q.** (A) Mid-level data fusion PCA score plot, PC1 vs PC3, with ROI of specimen 65 projected (black squares). The points annotated correspond to specific ROIs indicated on the H&E stained tissue in (B). Score plot symbols: Grey matter (green), white matter (blue), and glioma (red). **Figure R.** (A) Midlevel data fusion PCA score plot, PC1 vs PC3, with ROI of specimen 24 projected (black squares). Score plot symbols: Grey matter (green), white matter (blue), and glioma (red). (B) ROI are annotated upon the H&E stained tissue section with accompanying selected ion images plotted in false-color with corresponding scale bar. Note, the regions of predominately normal grey (*m/z* 834) and white (*m/z* 888) contain approximately 10% tumor cells. **Figure S.** (A) Negative and (B) positive ion mode lipid profile from a specimen 70 that was effaced, morphologically. The spectra appear similar to that of white matter (via *m/z* 888 detection) and suggest the background parenchyma is white matter.
(DOCX)

S1 Video 3D PCA score plot of fused positive and negative mode spectra (*m/z* 700–1000) for grey matter (green), white matter (blue), and glioma (red). (AVI)
