# Supplementary material for: Differential Lipid Profiles of Normal Human Brain Matter and Gliomas by Positive and Negative Mode Desorption Electrospray Ionization – Mass Spectrometry Imaging
Source: PLoS One. 2016 Sep 22;11(9):e0163180. doi: 10.1371/journal.pone.0163180 (PMC5033406; doi:10.1371/journal.pone.0163180)
Supplement: S1 File — Table A. HRMS and MS/MS data for selected ions detected in positive mode DESI-MSI of brain tissue and glioma specimens. Exact mass measurements were searched in the METLIN database (https://metlin.scripps.edu/metabo_search_alt2.php). MS/MS spectra were examined for expected losses from membrane lipid head groups such as 59 (trimethylamine) and 183 (phosphocholine) for PCs and SMs [45,46]; 162 (dehydrated C6 sugar), 180 (C6 sugar) for GalCer [47]; 43 and 141 (phosphoethanolamine) for PE [48,49]. Molecular species are consistent with previously published results in which lipid profiles from a small set of human astrocytoma specimens were obtained [21]. Plasmalogens were also detected at certain m/z values and supported with MS/MS experiments. Table B. Positive ion mode PCA-LDA confusion matrix (six principal components, five deletion groups) for grey matter, white matter, and glioma with calculated sensitivity and specificity for each class. Table C. Negative ion mode PCA-LDA confusion matrix (six principal components, five deletion groups) for grey matter, white matter, and glioma with calculated sensitivity and specificity for each class. Figure A. (A) Negative-mode DESI-MS spectra of ROI removed due to OCT signal (B) detected in the positive-mode. OCT is a polymeric material, principally polyvinyl alcohol, which is readily ionized in the positive ion mode, easily recognized by polymeric peaks separated by 22 mass-to-charge units (e.g., m/z 708.7, 730.7, and 752.7; m/z 715.1 and 737.1; m/z 722.7 and 744.7). Figure B. High resolution mass spectrum obtained from specimen CGY, composed of normal white and grey matter, in the positive ion mode A) m/z range 748–811. B) m/z range 828–852. Figure C. (A) MS2 product ion spectrum of m/z 750. (B) MS3 product ion spectrum of m/z 588 ([GalCer(d36:1) + Na– 162]+. (C) MS3 product ion spectrum of m/z 707 ([PE(P-36:2) + Na– 43]+. (D) MS2 product ion spectrum of m/z 772. (E) MS3 product ion spectrum of m/z 713 ([PC 32:0 + K– 59]+. ( [file pone.0163180.s001.docx]

Differential Lipid Profiles of Normal Human Brain Matter and Gliomas by Positive and Negative Mode Desorption Electrospray Ionization – Mass Spectrometry Imaging

Alan K. Jarmusch^1¶^, Clint M. Alfaro^1¶^, Valentina Pirro^1^, Eyas M. Hattab^2^, Aaron A. Cohen-Gadol^3^, and R. Graham Cooks^1*^

^1^Department of Chemistry and Center for Analytical Instrument Development, Purdue University, West Lafayette, IN 47907

^2^Department of Pathology and Laboratory Medicine, University of Louisville School of Medicine, Louisville, KY 40292

^3^Department of Neurological Surgery, Indiana University School of Medicine, Indianapolis, IN 46202

* corresponding author

Email: cooks@purdue.edu

^¶^ authors contributed equally

**Supporting Information**

**Experimental**

**Specimens**

Banked (frozen and unfixed) tissue specimens from 42 human subjects, obtained from the Biorepository of Methodist Research Institute (Indianapolis, IN, USA), suspected to be normal brain parenchyma, infiltrated tissue, or gliomas were included in this study. One specimen from three subjects was excluded *a priori* due to the presence of significant amounts of necrosis; therefore, the final number of tissue specimens and subjects included in the study was 39. Specimens were purchased after Purdue IRB determined IRB review was not required (#1410015344). Specimens were embedded in Optimal Cutting Temperature polymer (Sakura Finetek, Torrance, CA), cryosectioned on a cryotome FSE (Thermo Scientific, San Jose, CA) into 15 μm thick sections, and thaw mounted onto glass microscope slides (Gold Seal UltraFrost Frosted Slides, Thermo Scientific, San Jose, CA). Tissue sections were stored at –80°C prior to analysis.

**DESI-MS analysis**

DESI-MSI was performed using a linear ion trap mass spectrometer, model Finnigan LTQ (Thermo Electron Corporation, USA). The instrument was modified in the following ways: a source override interlock adapter, an external cable for the application of high voltage, and an extended ion transfer capillary; additional details can be found in the supplementary information appendix of Jarmusch et al 2016 [[1](#_ENREF_1)]. The mass spectrometer was tuned for maximum transmission of *m/z* 786 (dioleoylphosphatidylcholine) and 735 (dipalmitoylphosphatidylcholine) in the negative and positive ion modes, respectively. Dimethylformamide-acetonitrile (1:1 *v/v*) was used for DESI-MS imaging to preserve tissue morphology for subsequent pathology [[2](#_ENREF_2)]. Dimethylformamide (DMF) and acetonitrile (ACN) were purchased from Mallinckrodt Chemicals and Sigma-Aldrich, respectively. Additional source parameters are as follows: solvent flow rate, 1.0 µL min^-1^; pressure of nitrogen gas, 160 PSI; applied high voltage, -5.0 kV negative mode and +4.5kV in the positive mode; incident angle, 52°; spray-to-surface distance, 2–3 mm; spray-to-inlet distance, 5–7 mm.

DESI-MSI was performed on tissue sections by affixing the glass microscope slides onto a custom two-dimensional precision moving stage. Images were collected in a series of rows by coordinating linear motion of the moving stage (431.034 µm s^-1^) with MS duty cycle. The MS duty cycle was defined by the scan parameters: *m/z* 200-1000, injection time of 500 ms with 1 microscan, and automatic gain control (AGC) was disabled. A lateral spatial resolution (“x”) of 250 µm was defined, upon completion of a row the moving stage resets to the original “x” position while stepping 250 µm in “y”. This process was repeated in order to acquire data from the entire tissue surface. Note that the lateral spatial resolution was not optimized beyond 250 μm; the appropriate choice of solvents and conditions (e.g., solvent flow rate, pneumatic pressure, and MS scan rate) can result in much lower lateral spatial resolutions (e.g., < 50 μm) [[3](#_ENREF_3)]. Each tissue section was analyzed twice, initially in the positive mode and subsequently in the negative ion mode. The moving stage was reset to the origin position between images, thus the positive and negative DESI-MS data from individual pixels were superimposable. The positive-mode was chosen first as ions detected were typically sodium or potassium adducts, and by imaging for positive ions first we minimized any changes in adduct formation due to prior analysis. Conversely, this was less of a concern in the negative mode as many of the ions were detected as deprotonated species; however, important chloride adducts of PCs were also detected (*m/z* 794).

Parameters for MS^n^ on the LTQ ion trap were: isolation width of *m/z* 0.7, q-value of 0.25, normalized collision energy of 35 arbitrary units, 2 microscans, and 250 ms maximum injection time. MS/MS spectra were recorded for several ions, selected for their importance in distinguishing tissue types based on loading plots resulting from principal component analysis (PCA), from representative normal brain matter and glioma specimens. MS^3^ was performed on ions derived from neutral losses of expected phospholipid head fragments from the precursor ions [[4](#_ENREF_4)].

High-resolution MS data were obtained at multiple spots from representative specimens of normal brain matter and glioma using an Orbitrap mass spectrometer (Thermo Exactive, San Jose, CA) to aid in lipid attribution. The Exactive was calibrated in both polarities prior to analysis. Exactive parameters were as follows: resolution was set to ultrahigh (100,000), AGC was set to ultimate mass accuracy (5*10^5^), max injection time was 500 ms, and DESI parameters were identical to those described previously. Data were acquired from *m/z* 500–1000. Resolution at *m/z* 798.5415 was calculated to be approximately 58,000 at full-width half-maximum using the m/Δm method. The measured *m/z* were searched on the Metlin database (https://metlin.scripps.edu/metabo_search_alt2.php) and most of the peaks provided multiple potential identifications within 5 ppm mass error.

**Pathology**

After DESI-MSI, the tissue sections were hematoxylin and eosin (H&E) stained, following a protocol recently described by Jarmusch et al [[1](#_ENREF_1)], and blindly evaluated by an expert pathologist (E.M.H.) for disease state (e.g., glioma), tumor grade, and tumor cell percentage. Histopathologic evaluation determined that a number of the specimens contained multiple regions with different pathologies.

**Data Analysis**

DESI-MSI data were acquired using Xcalibur 2.0 (.raw) and converted into .mzXML files using msConvert (http://proteowizard.sourceforge.net/downloads.shtml). The .mzXML files were imported into MATLAB (MathWorks, Natick, MA) and used to create hyperspectral datacubes (spatial information and spectral information). Regions of interest (ROI) were selected in MATLAB based on histopathologic review, each ROI selection is the average spectrum of 1 mm (4 pixels) x 1 mm (4 pixels). Additional details can be found in the supplementary material of Jarmusch et al [[1](#_ENREF_1)]. The set of tissue sections used provided 585 ROI; 32 selections were removed as they contained significant amounts of ions related to OCT (see *Specimens*) in the spectra (Fig A). The remaining selections were examined by PCA from *m/z* 700 - 1000; the exclusion of lower *m/z* values improved separation as the lower mass-to-charge region were more variable in intensity and displayed more background signal.

PCA, using the nonlinear iterative partial least squares (NIPALS) algorithm, was used to explore the DESI-MSI data in an unsupervised fashion and to visualize groupings of ROI based on chemical similarity. PCA was performed on mean-centered, standard normal variate (SNV) transformed MS spectra. SNV corrects for both baseline shifts and global intensity variations [[5](#_ENREF_5)]. No background signal correction, smoothing filters, or data binning was applied. PCA was also performed after mid-level data fusion. The first round of PCA acts as an unsupervised latent-variable compression technique, informative features were included from the raw signals of each block (i.e., negative lipid profile and positive lipid profile) by PCA, individually, and then combined into a new data set of principal component score, and then processed again by PCA. The merged principal component score data set is autoscaled [[6](#_ENREF_6)].

Linear discriminant analysis (LDA) was performed as a supervised discriminant classification technique on the PCA-compressed dataset. LDA was applied after unsupervised data compression using PCA on the negative ion mode data, positive ion mode data, and the midlevel fusion data. Model validation (i.e., evaluation of the predictive ability of the model) was performed with cross-validation using five deletion groups. The cross-validation confusion matrix shows how many samples belonging to a certain category were correctly/incorrectly assigned by the classification rule to that category. Sensitivity (i.e., measure of the proportion of positives that are correctly identified as such) and specificity (i.e., measure of the proportion of negatives that are correctly identified as such) were calculated by conventional methods.

**Figures and Tables**

**Table A.** HRMS and MS/MS data for selected ions detected in positive mode DESI-MSI of brain tissue and glioma specimens. Exact mass measurements were searched in the METLIN database (<https://metlin.scripps.edu/metabo_search_alt2.php>). MS/MS spectra were examined for expected losses from membrane lipid head groups such as 59 (trimethylamine) and 183 (phosphocholine) for PCs and SMs [[7](#_ENREF_7),[8](#_ENREF_8)]; 162 (dehydrated C_6_ sugar), 180 (C_6_ sugar) for GalCer [[4](#_ENREF_4)]; 43 and 141 (phosphoethanolamine) for PE [[9](#_ENREF_9),[10](#_ENREF_10)]. Molecular species are consistent with previously published results from our group in which lipid profiles from a small set of human astrocytoma specimens were obtained [[11](#_ENREF_11)]. Plasmalogens were also detected at certain *m/z* values and supported with MS/MS experiments.

| Tentative Ion Identification | Theoretical Mass (Monoisotopic) | Measured Mass | Mass Error (ppm) | Major MS^2^ Fragment Ions | MS^3^ Fragment Ions |
| --- | --- | --- | --- | --- | --- |
| [PE(P-36:2)+Na]^+^ | 750.5408 | 750.5407 | -0.1865 | 707 (-43) | 707->609, 587, 441 |
| [GalCer(d36:1)+Na]^+^ | 750.5854 | 750.5855 | 0.1332 | 732 (-18), 588 (-162), 570 (-180) | 588->570 |
| [PC(32:1)+Na]^+^ | 754.5357 | 754.5362 | 0.6627 | 695 (-59), 571 (-183) | 695->571, 549 |
| [PC(32:0)+Na]^+^ | 756.5514 | 756.5530 | 2.1149 | 697 (-59), 573 (-183) | 697->573, 551 |
| [PC(32:0)+K]^+^ | 772.5253 | 772.5281 | 3.6245 | 713 (-59) | 713->589, 551 |
| [PE(P-38:5)+Na]^+^ | 772.5252 | 772.5281 | 3.7125 | 729 (-43) | 729->631, 489 |
| [PC(34:1)+Na]^+^ | 782.5670 | 782.5679 | 1.1705 | 723 (-59) | 723->599, 577 |
| [PC(34:1)+K]^+^ | 798.5410 | 798.5415 | 0.6224 | 739 (-59), 615 (-183) | 739->615, 577 |
| [PE(P-40:6)+Na]^+^ | 798.5408 | 798.5415 | 0.8728 | 755 (-43) | 755->657, 487 |
| [PC(36:2)+Na]^+^ | 808.5827 | 808.5851 | 3.0226 | 749 (-59), 625 (-183) | 749->625, 603 |
| [GalCer(d32:2)+Na]^+^ | 832.6636 | 832.6642 | 0.6701 | 814 (-18), 670 (-162), 652  (-180) | 670->652 |
| [GalCer(d32:2)+K]^+^ | 848.6376 | 848.6390 | -1.5979 | 686 (-162), 668 (-180) | 686->668 |
|  |  |  |  |  |  |

**Table B.** Positive ion mode PCA-LDA confusion matrix (six principal components, five deletion groups) for grey matter, white matter, and glioma with calculated sensitivity and specificity for each class.

|  |  | Histopathology | | |
| --- | --- | --- | --- | --- |
|  |  | Grey matter | White matter | Glioma |
| DESI | Grey matter | 216 | 9 | 13 |
|  | White matter | 1 | 88 | 0 |
|  | Glioma | 6 | 1 | 172 |
|  | Sensitivity (%) | 96.9 | 89.8 | 93.0 |
|  | Specificity (%) | 92.2 | 99.7 | 97.7 |

**Table C.** Negative ion mode PCA-LDA confusion matrix (six principal components, five deletion groups) for grey matter, white matter, and glioma with calculated sensitivity and specificity for each class.

|  |  | Histopathology | | |
| --- | --- | --- | --- | --- |
|  |  | Grey matter | White matter | Glioma |
| DESI | Grey matter | 219 | 2 | 1 |
|  | White matter | 4 | 94 | 21 |
|  | Glioma | 0 | 2 | 163 |
|  | Sensitivity (%) | 98.2 | 95.9 | 88.1 |
|  | Specificity (%) | 98.8 | 93.9 | 99.4 |


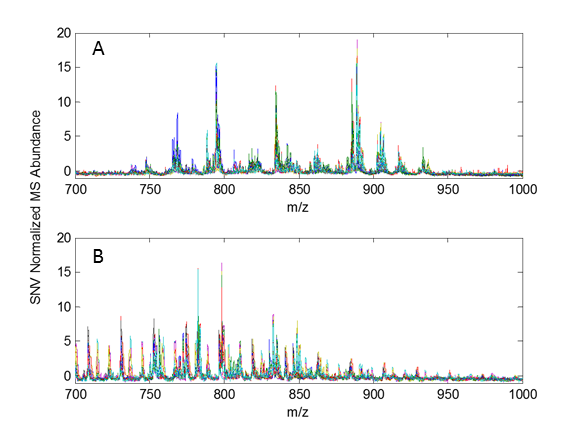


**Figure A.** (A) Negative-mode DESI-MS spectra of ROI removed due to OCT signal (B) detected in the positive-mode. OCT is a polymeric material, principally polyvinyl alcohol, which is readily ionized in the positive ion mode, easily recognized by polymeric peaks separated by 22 mass-to-charge units (*e.g., m/z* 708.7, 730.7, and 752.7; *m/z* 715.1 and 737.1; *m/z* 722.7 and 744.7).


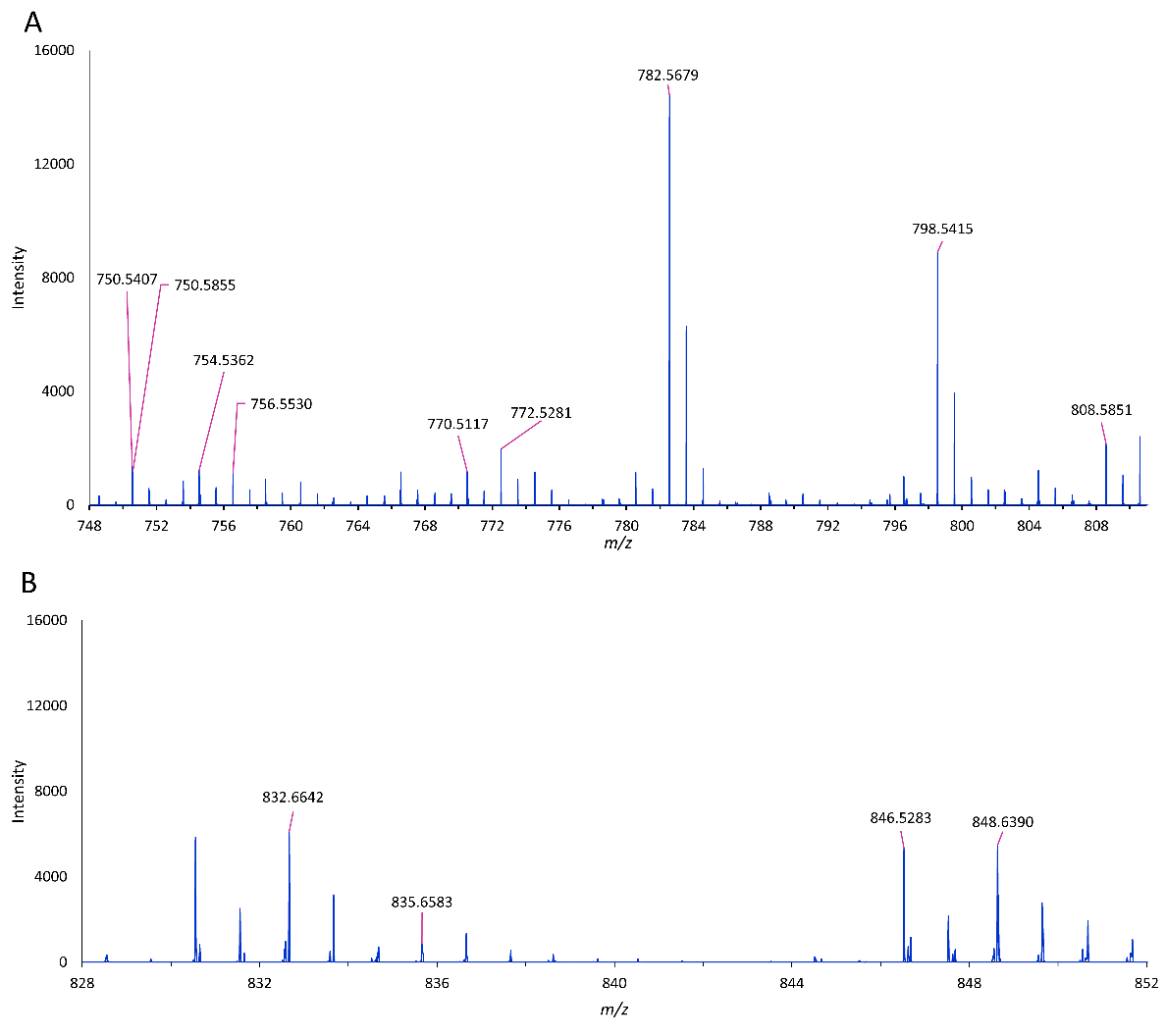


**Figure B.** High resolution mass spectrum obtained from specimen CGY, composed of normal white and grey matter, in the positive ion mode A) *m/z* range 748-811. B) *m/z* range 828-852.


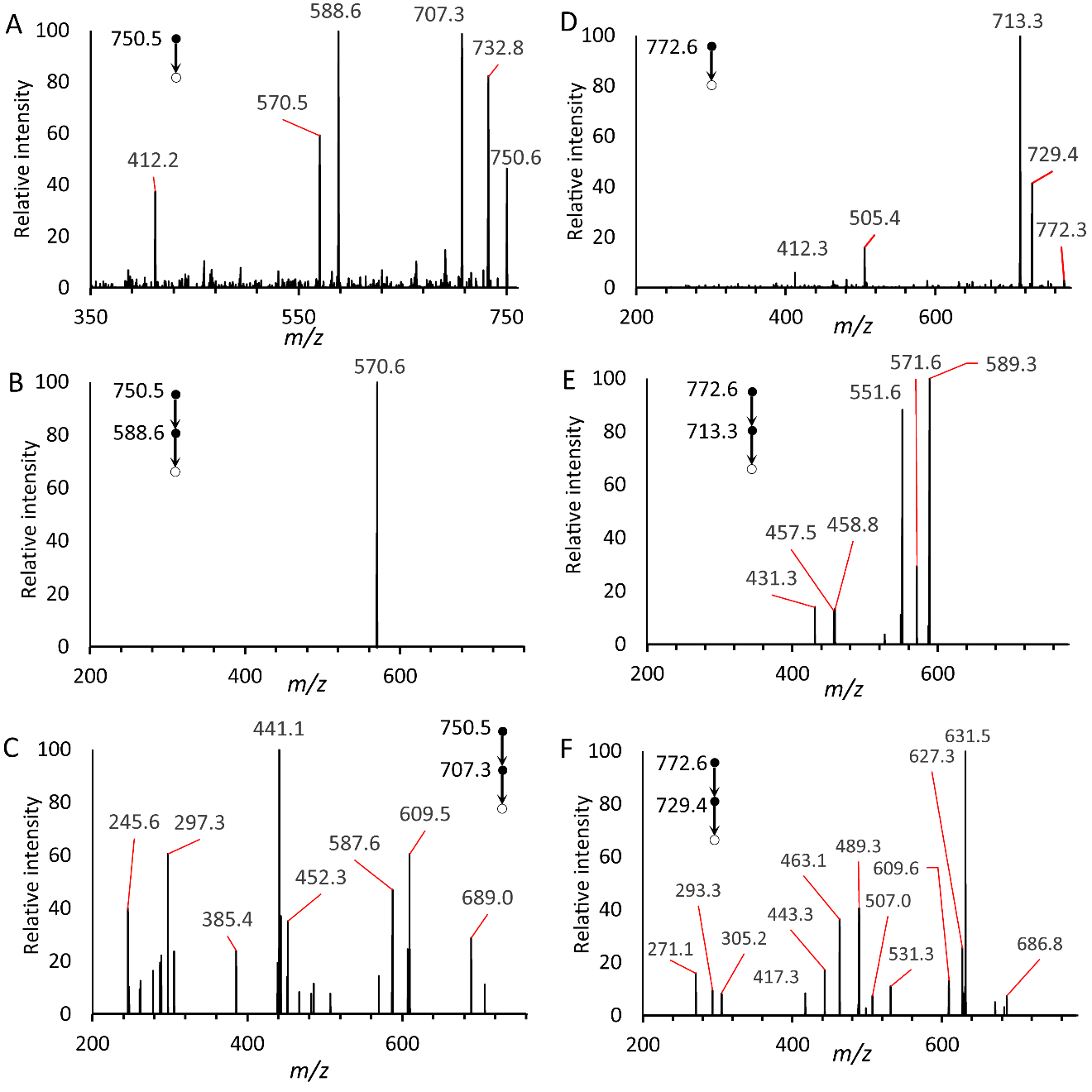


**Figure C.** (A)MS^2^ product ion spectrum of *m/z* 750. (B) MS^3^ product ion spectrum of *m/z* 588 ([GalCer(d36:1) + Na - 162]^+^. (C) MS^3^ product ion spectrum of *m/z* 707 ([PE(P-36:2) + Na - 43]^+^. (D) MS^2^ product ion spectrum of *m/z* 772. (E) MS^3^ product ion spectrum of *m/z* 713 ([PC 32:0 + K - 59]^+^. (F) MS^3^ product ion spectrum of *m/z* 729 ([PE(P-38:5) + Na - 43]^+^.


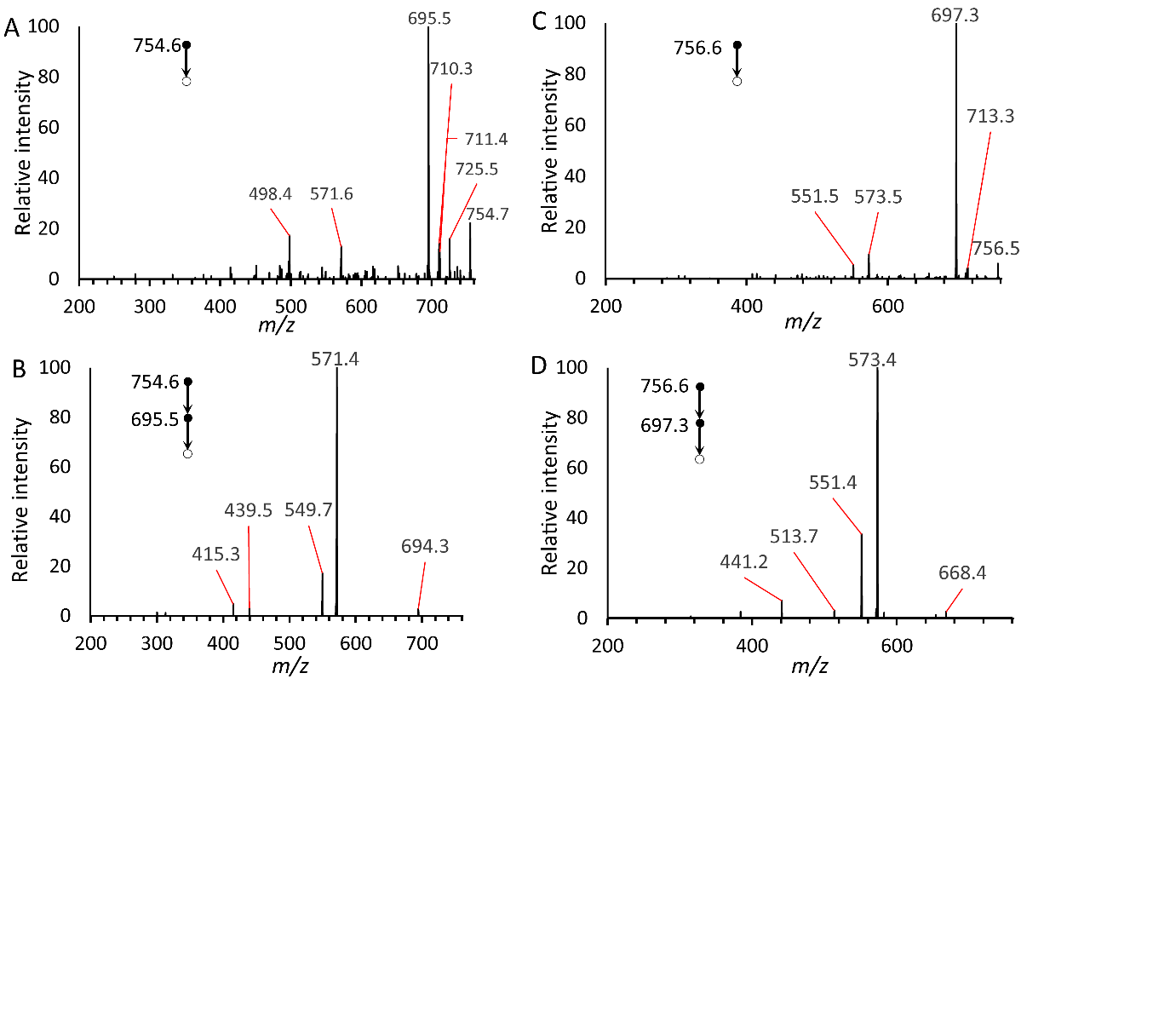


**Figure D.** (A)MS^2^ product ion spectrum of *m/z* 754. (B) MS^3^ product ion spectrum of *m/z* 695 ([PC 32:1 + Na - 59]^+^). ( C) MS^2^ product ion spectrum of *m/z* 756. (D) MS^3^ product ion spectrum of *m/z* 697 ([PC 32:0 + Na - 59]^+^).


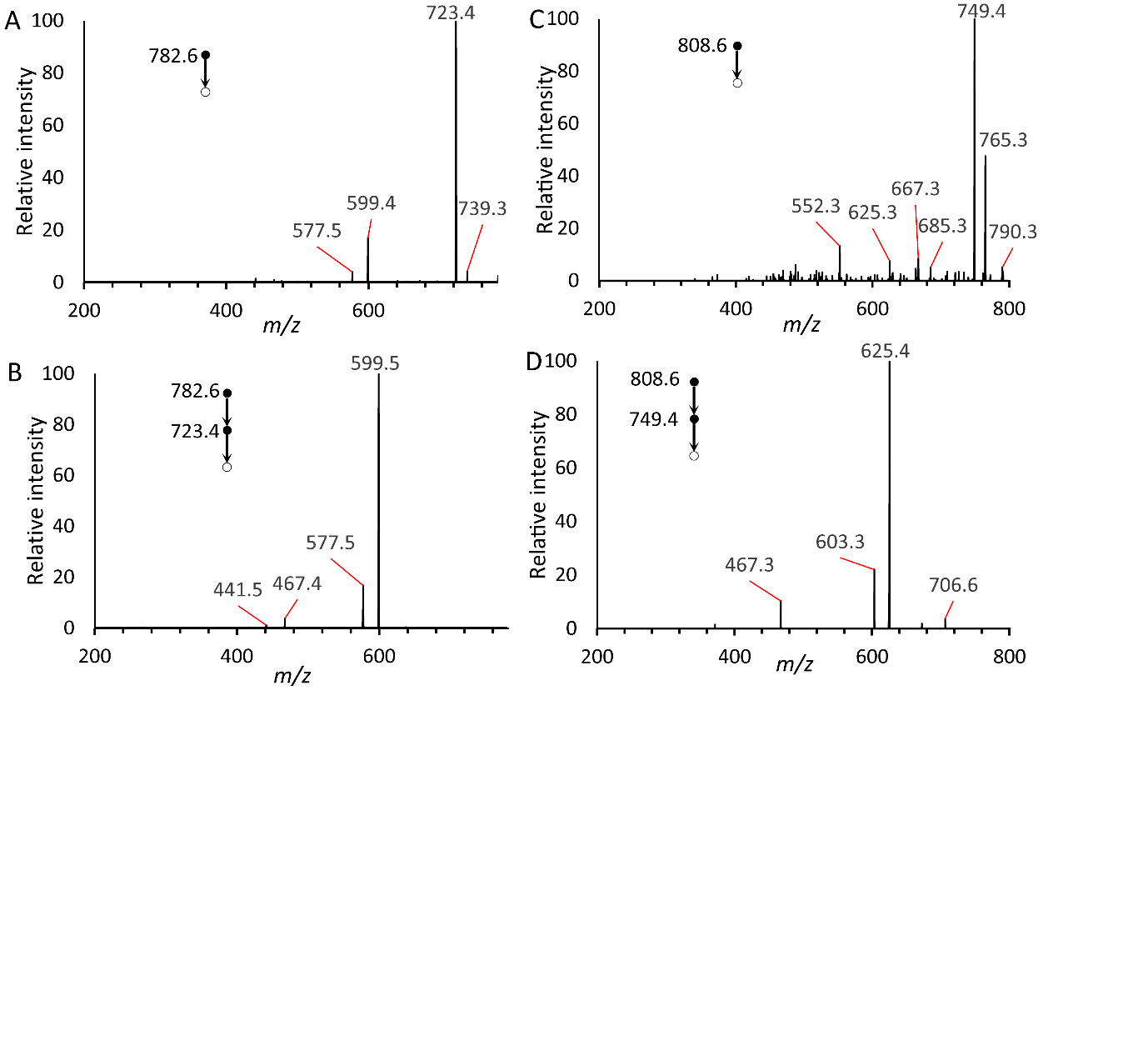


**Figure E.** (A)MS^2^ product ion spectrum of *m/z* 782. (B) MS^3^ product ion spectrum of *m/z* 723 ([PC 34:1 + Na - 59]^+^). ( C) MS^2^ product ion spectrum of *m/z* 808. (D) MS^3^ product ion spectrum of *m/z* 749 ([PC 36:2 + Na - 59]^+^).


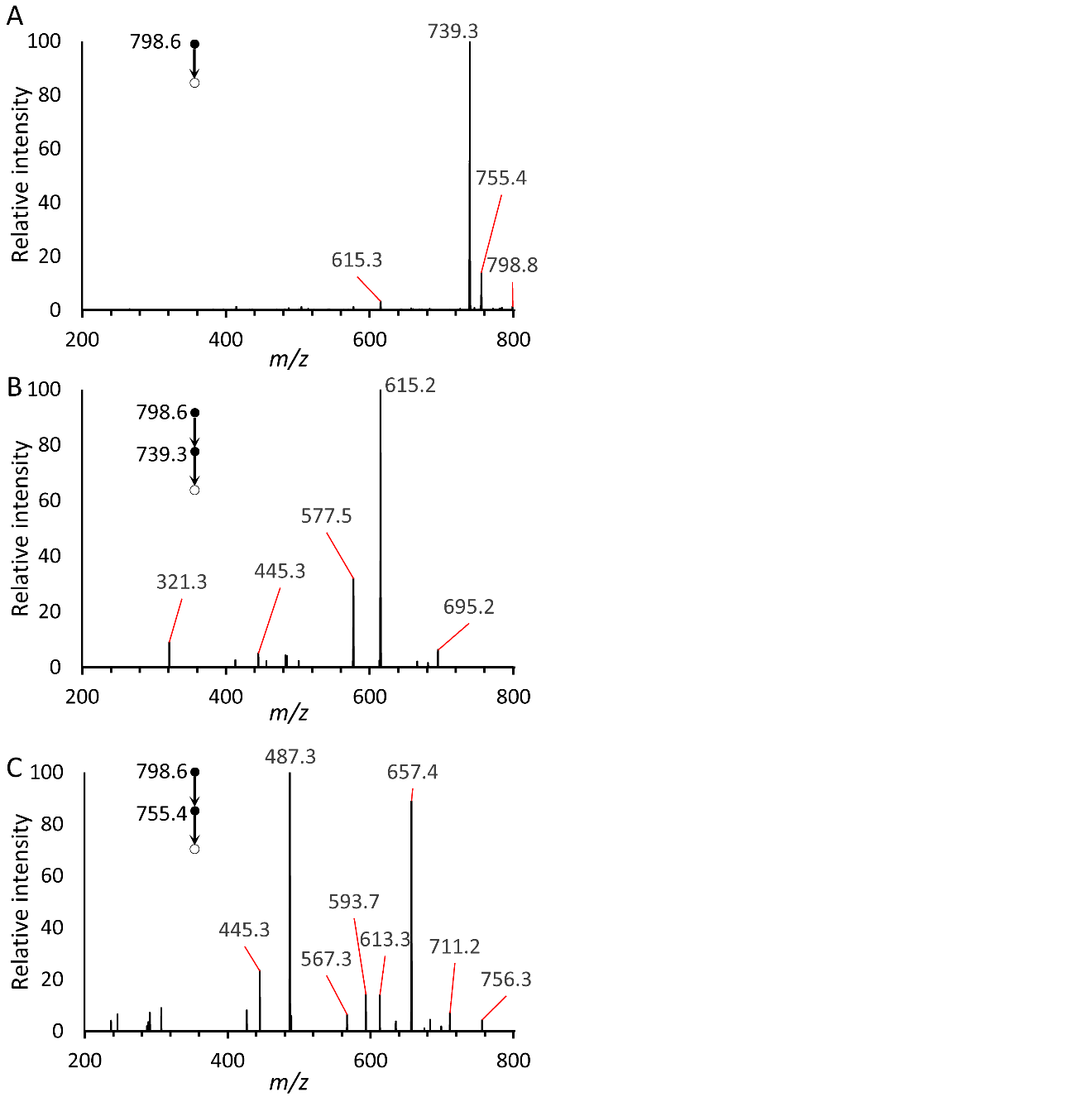


**Figure F.** (A)MS^2^ product ion spectrum of *m/z* 798. (B) MS^3^ product ion spectrum of *m/z* 739 ([PC 34:1 + K - 59]^+^). (C) MS^3^ product ion spectrum of *m/z* 755 ([PE(P-40:6) + Na - 43]^+^).


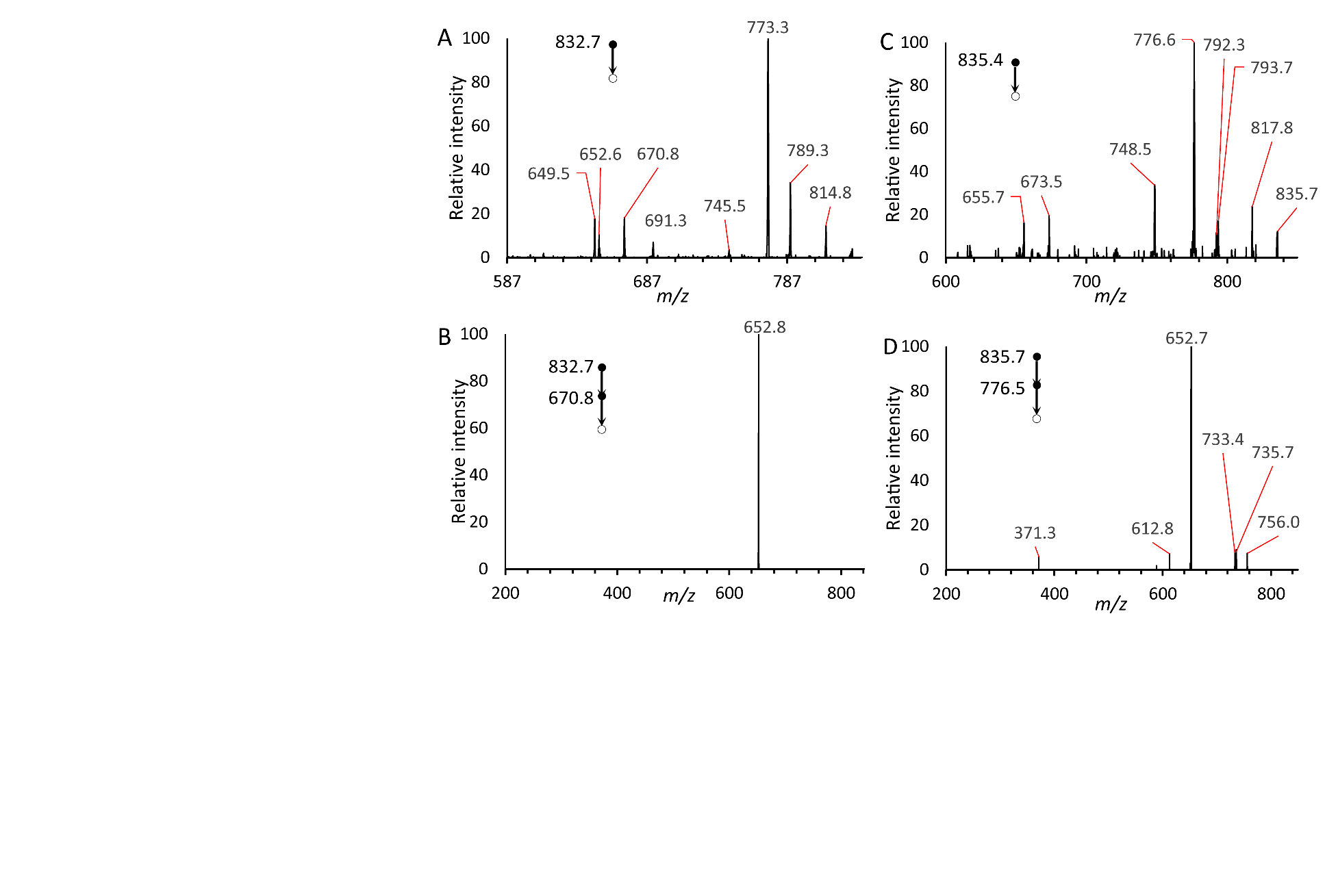


**Figure G.** (A)MS^2^ product ion spectrum of *m/z* 832. (B) MS^3^ product ion spectrum of *m/z* 670 ([GalCer(d32:2) + Na - 162]^+^). (C) MS^2^ product ion spectrum of *m/z* 835. (D) MS^3^ product ion spectrum of *m/z* 776.


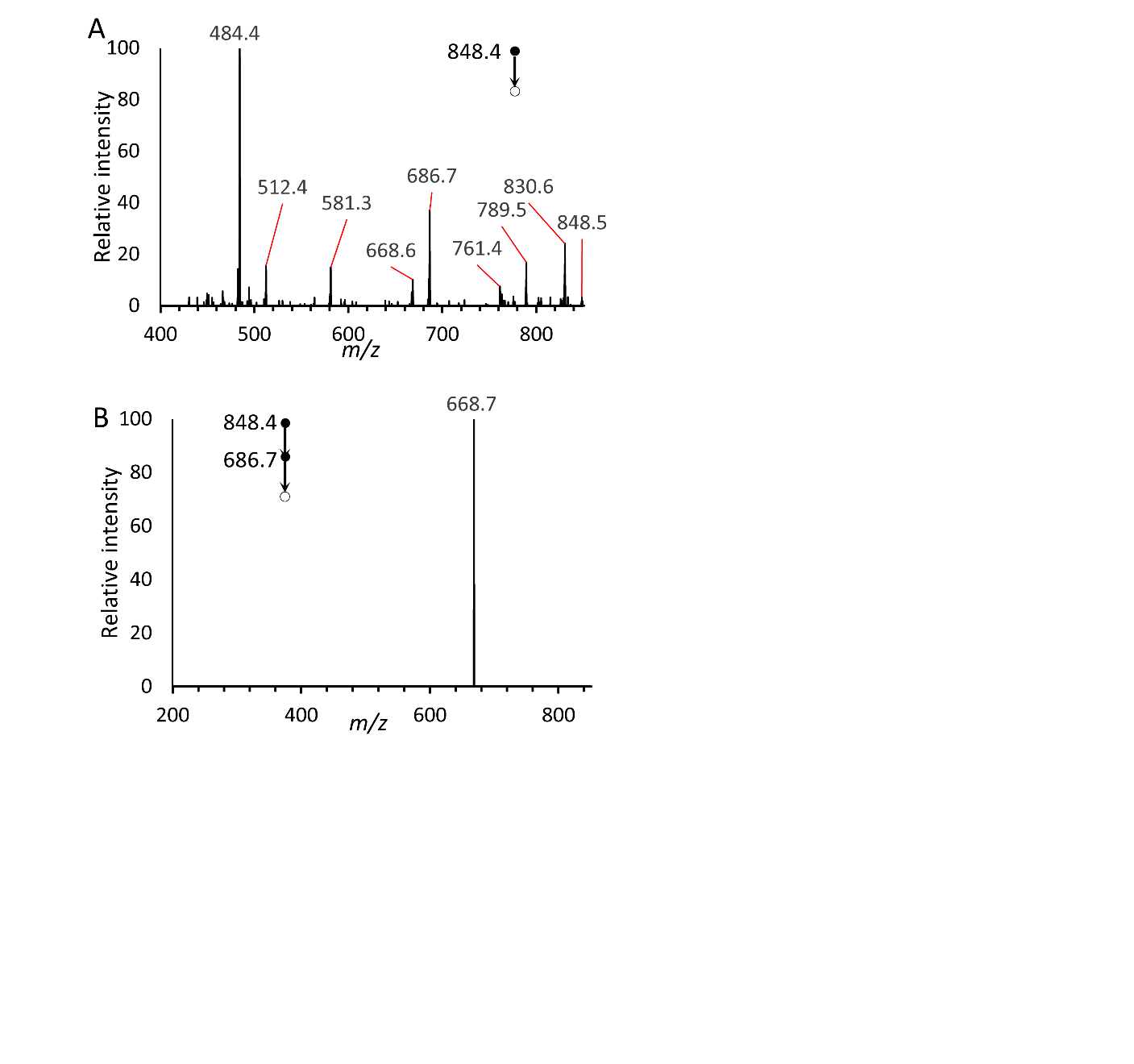


**Figure H.** (A)MS^2^ product ion spectrum of *m/z* 848. (B) MS^3^ product ion spectrum of *m/z* 686 ([GalCer(d32:2) + K - 162]^+^).


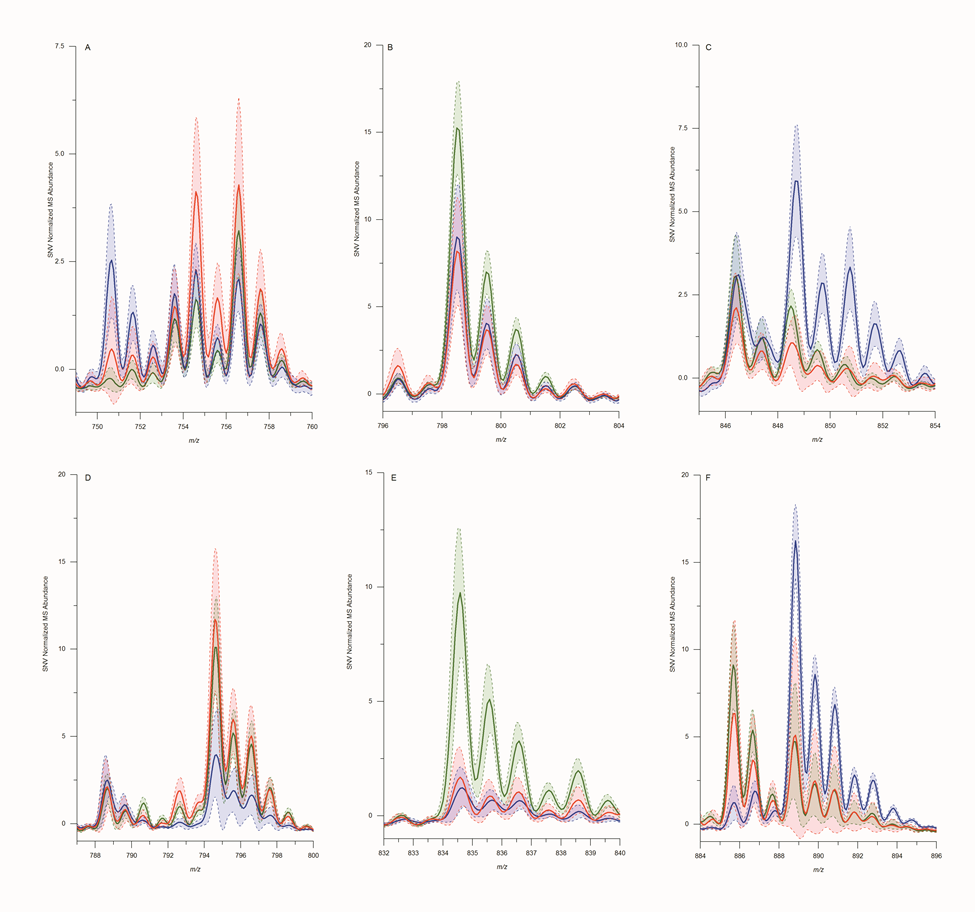


**Figure I.** (A-C) Selected *m/z* regions of the positive ion mode lipid profile displaying the mean (solid line) and standard deviation (dotted line and filled area) for grey matter (green), white matter (blue), and glioma (red). (D-E) Selected *m/z* regions of the negative ion mode lipid profile. The mean (solid line) and standard deviation (dotted line with filled area) are displayed for grey matter (green), white matter (blue), and glioma (red).


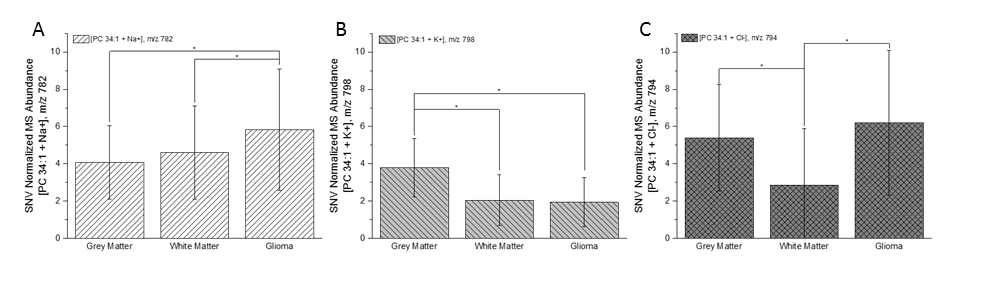


**Figure J.** Bar graph of the SNV normalized MS abundance of (A) *m/z* 782, (B) *m/z* 798, and (C) *m/z* 794, associated with different adducts of PC 34:1 (sodium, potassium, and chloride respectively), displaying the mean and standard deviation per class: grey matter (N=223), white matter (N=98), and glioma (N=200). Statistical significance indicated (Kruskal-Wallis, 95% confidence) by a single asterisk (*). Overall, the differences in abundance were not predictive by themselves due to high variances, while differences in some mean values were statistically significant at 95% confidence (*e.g., m/z* 782, grey matter and glioma) by Kruskal-Wallis.


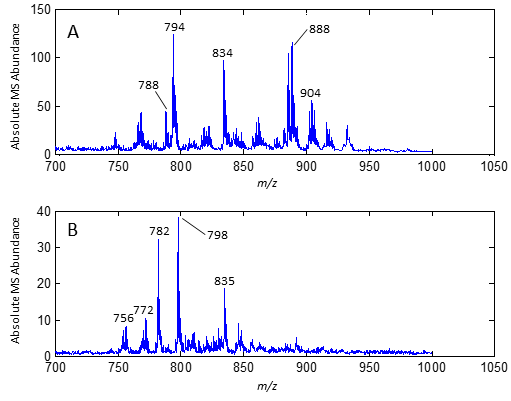


**Figure K.** Illustrative (A) negative and (B) positive ion mode DESI-MS spectra from a ROI of mixed grey and white matter composition. Both *m/z* 834 and 888 are abundant in the negative ion mode. Similarly, the positive mode lipid profile appears to be a combination of grey and white matter.


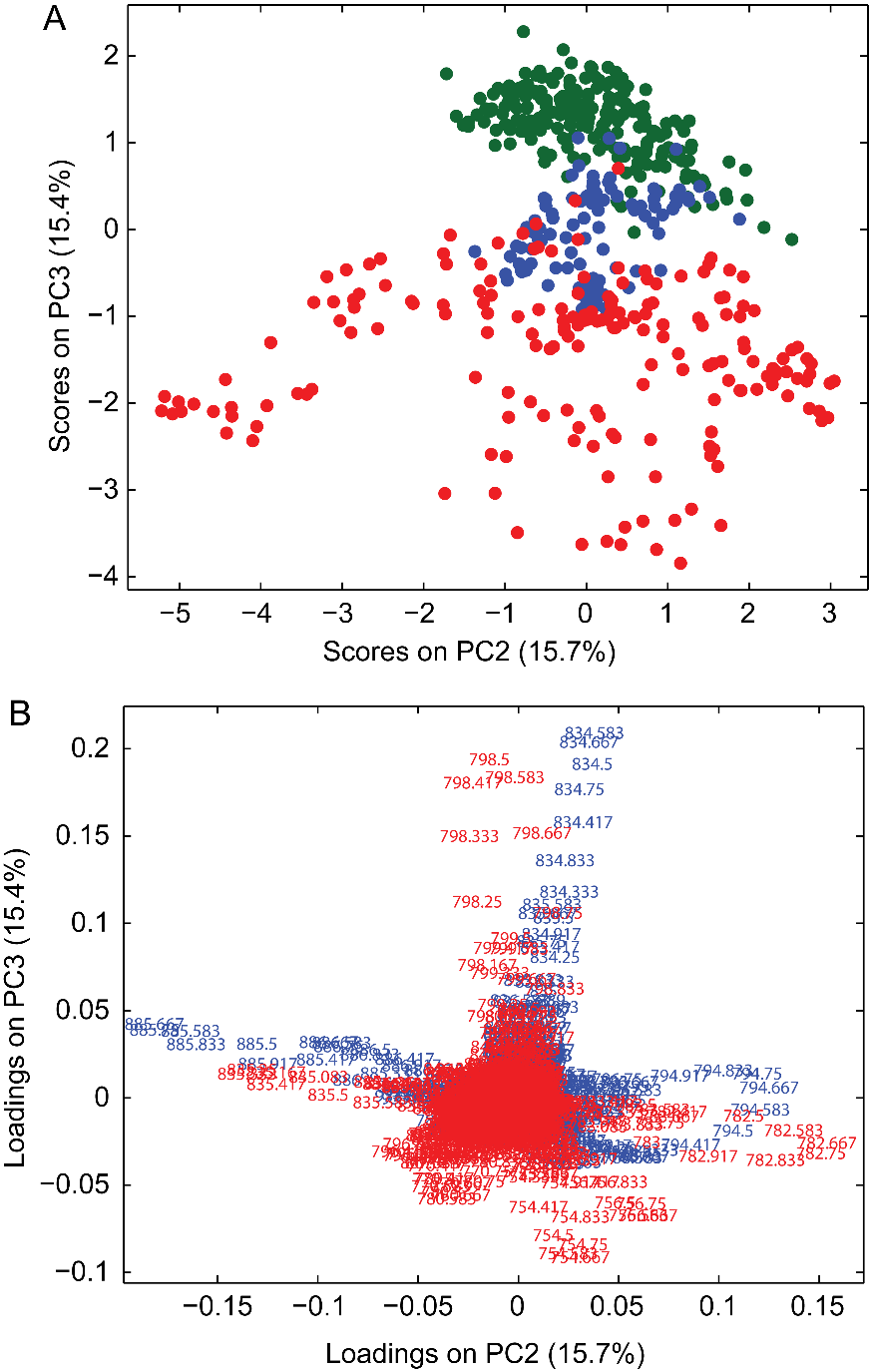


**Figure L.** (A) Mid-level data fusion PCA score plot, PC2 vs PC3, and (B) loading plots. Score plot symbols: Grey matter (green), white matter (blue), and glioma (red). Loading plot values: negative-mode (blue) and positive-mode (red).





**Figure M.** (A-B) Negative and positive mode average of the white matter associated glioma subgroup, respectively. (C) Negative mode average of the grey matter associated glioma subgroup. (D) Positive mode average of the grey matter associated glioma subgroup. Major ions are annotated.


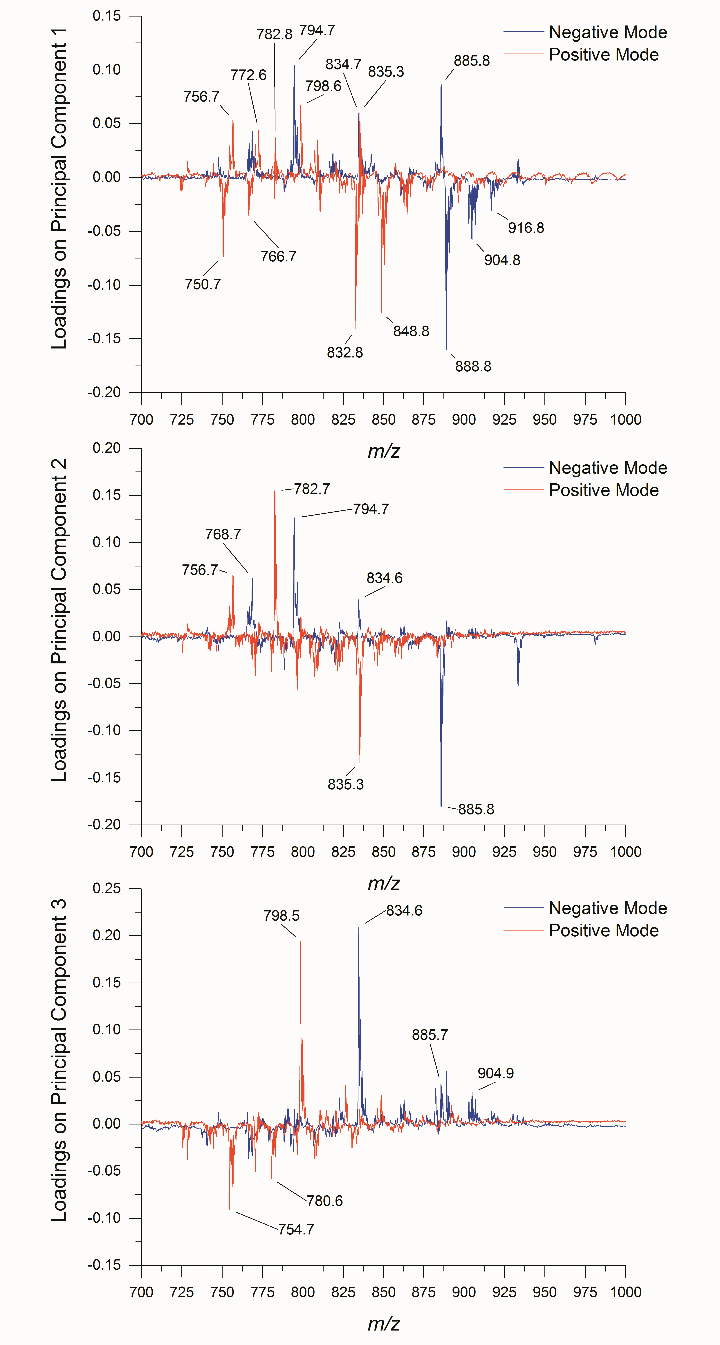


**Figure N.** Mid-level fusion PCA loading values of specific ions, negative mode (blue) and positive mode (red), on principal components 1-3 with *m/z* annotated.


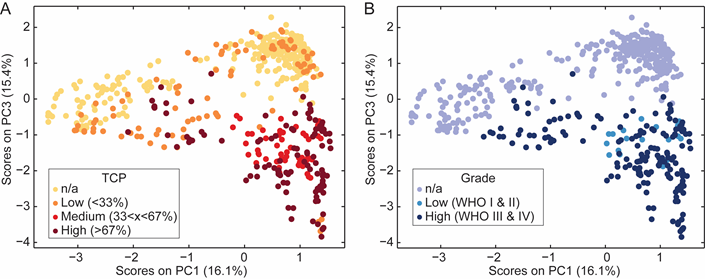


**Figure O.** (A) PCA score plot, PC1 vs PC3, for tumor cell percentage (TCP): n/a (yellow), normal grey or white matter; low (orange), <33%; medium (red), 33%<x<67%; high (dark red), >67%. (B) PCA score plot for glioma grade: n/a (blue heather), normal grey or white matter; low grade (light blue), glioma WHO grade I or II; high grade (dark blue), glioma WHO grade III or IV.


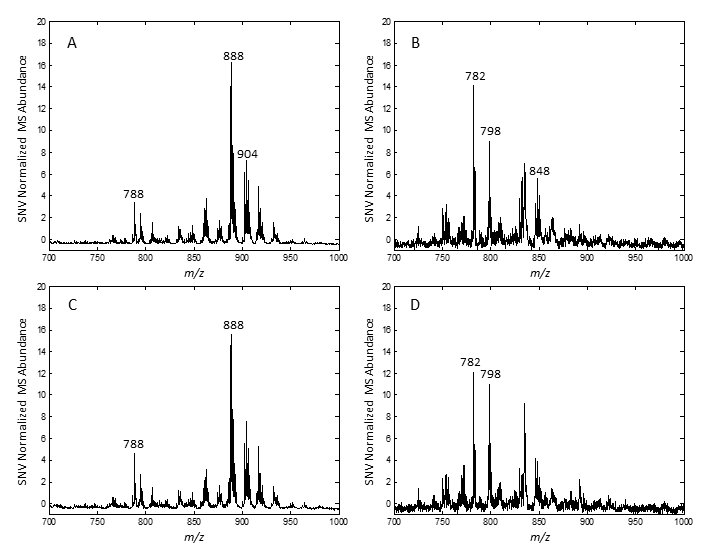


**Figure P.** (A) Negative and (B) positive ion mode lipid profiles of ROI #1 with 40% TCP. (C) Negative and (D) positive ion mode lipid profiles of ROI #5 with 60% TCP. Note, that the 40% TCP spectra look more reminiscent of normal white matter while the 60% TCP spectra appear more like the white matter associate glioma subgroup (*e.g.,* altered ratio between *m/z* 794 and *m/z* 888 in the negative ion mode and altered ratio of *m/z* 798 and *m/z* 848 in the positive mode).


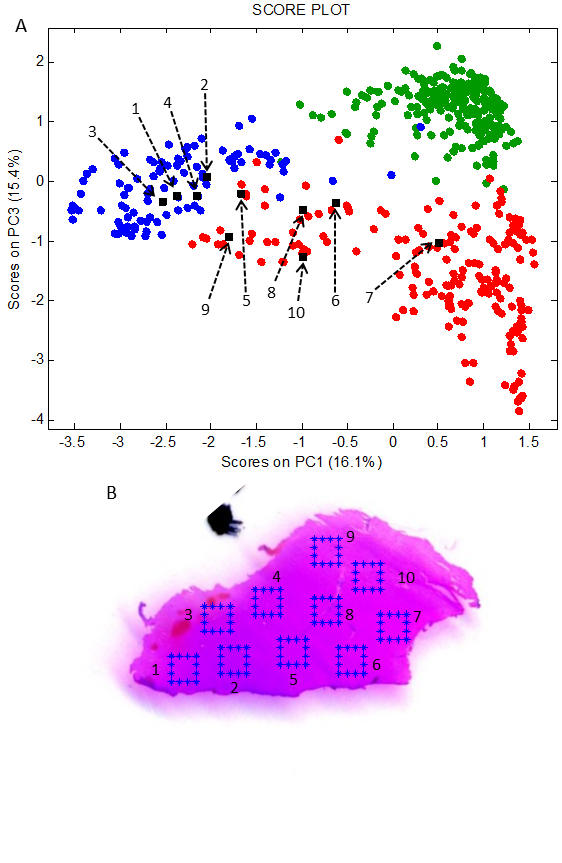


**Figure Q.** (A) Mid-level data fusion PCA score plot, PC1 vs PC3, with ROI of specimen 65 projected (black squares). The points annotated correspond to specific ROIs indicated on the H&E stained tissue in (B). Score plot symbols: Grey matter (green), white matter (blue), and glioma (red).


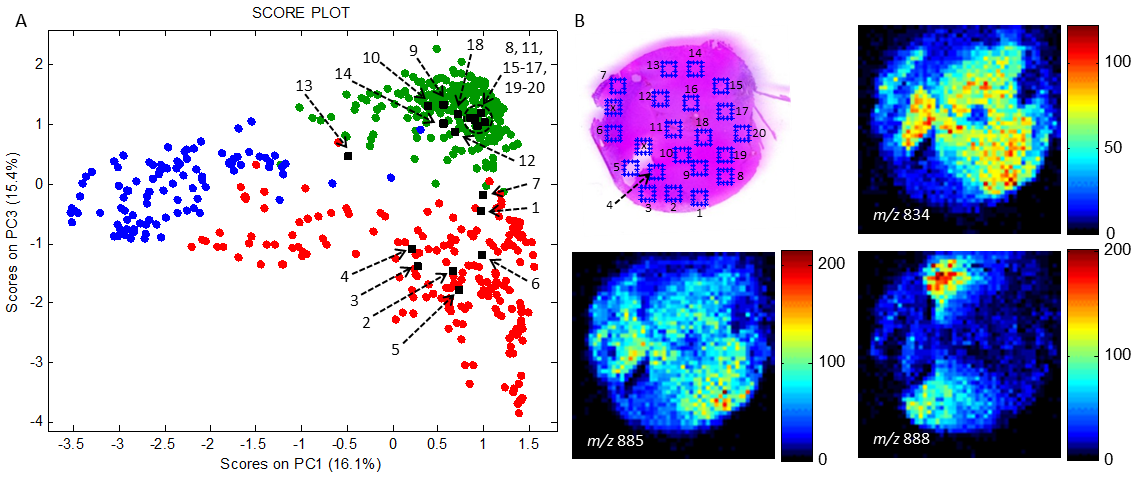


**Figure R.** (A) Mid-level data fusion PCA score plot, PC1 vs PC3, with ROI of specimen 24 projected (black squares). Score plot symbols: Grey matter (green), white matter (blue), and glioma (red). (B) ROI are annotated upon the H&E stained tissue section with accompanying selected ion images plotted in false-color with corresponding scale bar. Note, the regions of predominately normal grey (*m/z* 834) and white (*m/z* 888) contain approximately 10% tumor cells.


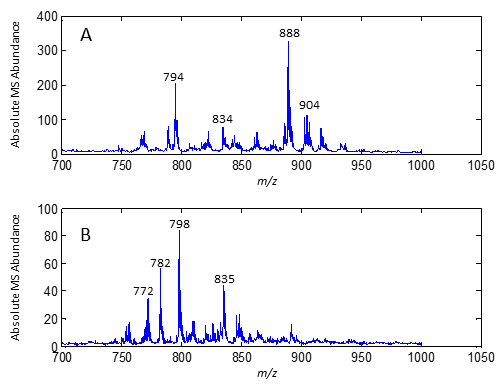


**Figure S.** (A) Negative and (B) positive ion mode lipid profile from a specimen 70 that was effaced, morphologically. The spectra appear similar to that of white matter (via *m/z* 888 detection) and suggest the background parenchyma is white matter.

**Video A.** 3D PCA score plot of fused positive and negative mode spectra (*m/z* 700 – 1000) for grey matter (green), white matter (blue), and glioma (red).

**SI References**

1. Jarmusch AK, Pirro V, Baird Z, Hattab EM, Cohen-Gadol AA, et al. (2016) Lipid and metabolite profiles of human brain tumors by desorption electrospray ionization-MS. Proceedings of the National Academy of Sciences: 201523306.

2. Eberlin LS, Ferreira CR, Dill AL, Ifa DR, Cheng L, et al. (2011) Nondestructive, histologically compatible tissue imaging by desorption electrospray ionization mass spectrometry. ChemBioChem 12: 2129-2132.

3. Campbell DI, Ferreira CR, Eberlin LS, Cooks RG (2012) Improved spatial resolution in the imaging of biological tissue using desorption electrospray ionization. Analytical and bioanalytical chemistry 404: 389-398.

4. Han XL, Gross RW (2005) Shotgun lipidomics: Electrospray ionization mass spectrometric analysis and quantitation of cellular lipidomes directly from crude extracts of biological samples. Mass Spectrometry Reviews 24: 367-412.

5. Fearn T (2009) The effect of spectral pre-treatments on interpretation. NIR news 20: 15-16.

6. Pirro V, Oliveri P, Ferreira CR, González-Serrano AF, Machaty Z, et al. (2014) Lipid characterization of individual porcine oocytes by dual mode DESI-MS and data fusion. Analytica Chimica Acta 848: 51-60.

7. Manicke NE, Wiseman JM, Ifa DR, Cooks RG (2008) Desorption electrospray ionization (DESI) mass Spectrometry and tandem mass spectrometry (MS/MS) of phospholipids and sphingolipids: Ionization, adduct formation, and fragmentation. Journal of the American Society for Mass Spectrometry 19: 531-543.

8. Hsu FF, Turk J (2003) Electrospray ionization/tandem quadrupole mass spectrometric studies on phosphatidylcholines: the fragmentation processes. J Am Soc Mass Spectrom 14: 352-363.

9. Hsu FF, Turk J (2000) Characterization of phosphatidylethanolamine as a lithiated adduct by triple quadrupole tandem mass spectrometry with electrospray ionization. J Mass Spectrom 35: 595-606.

10. Brügger B, Erben G, Sandhoff R, Wieland FT, Lehmann WD (1997) Quantitative analysis of biological membrane lipids at the low picomole level by nano-electrospray ionization tandem mass spectrometry. Proc Natl Acad Sci U S A 94: 2339-2344.

11. Eberlin LS, Dill AL, Golby AJ, Ligon KL, Wiseman JM, et al. (2010) Discrimination of human astrocytoma subtypes by lipid analysis using desorption electrospray ionization imaging mass spectrometry. Angew Chem Int Ed Engl 49: 5953-5956.
